# Loss of thymidine kinase 1 inhibits lung cancer growth and metastatic attributes by reducing GDF15 expression

**DOI:** 10.1371/journal.pgen.1008439

**Published:** 2019-10-07

**Authors:** Parmanand Malvi, Radoslav Janostiak, Arvindhan Nagarajan, Guoping Cai, Narendra Wajapeyee

**Affiliations:** 1 Department of Biochemistry and Molecular Genetics, University of Alabama at Birmingham, Birmingham, Alabama, United States of America; 2 Department of Pathology, Yale University School of Medicine, New Haven, Connecticut, United States of America; National Cancer Institute, UNITED STATES

## Abstract

Metabolic alterations that are critical for cancer cell growth and metastasis are one of the key hallmarks of cancer. Here, we show that thymidine kinase 1 (TK1) is significantly overexpressed in tumor samples from lung adenocarcinoma (LUAD) patients relative to normal controls, and this TK1 overexpression is associated with significantly reduced overall survival and cancer recurrence. Genetic knockdown of *TK1* with short hairpin RNAs (shRNAs) inhibits both the growth and metastatic attributes of LUAD cells in culture and in mice. We further show that transcriptional overexpression of TK1 in LUAD cells is driven, in part, by MAP kinase pathway in a transcription factor MAZ dependent manner. Using targeted and gene expression profiling-based approaches, we then show that loss of TK1 in LUAD cells results in reduced Rho GTPase activity and reduced expression of growth and differentiation factor 15 (GDF15). Furthermore, ectopic expression of GDF15 can partially rescue TK1 knockdown-induced LUAD growth and metastasis inhibition, confirming its important role as a downstream mediator of TK1 function in LUAD. Collectively, our findings demonstrate that TK1 facilitates LUAD tumor and metastatic growth and represents a target for LUAD therapy.

## Author summary

Thymidine kinase 1 (TK1) is overexpressed and associated with poor prognosis in a number of different cancers. However, despite these data suggesting an important role for TK1 in cancer pathogenesis, no study thus far has analyzed the functional effect of TK1 inhibition on tumor growth and metastasis. In this study, we performed TK1 knockdown and found that this protein is necessary for lung adenocarcinoma (LUAD) tumor growth and metastasis. Notably, inhibition of another nucleotide kinase, deoxycytidine kinase (DCK), had no effect on LUAD tumor growth and metastatic attributes. We therefore performed experiments to determine if the TK1 mechanism of action in cancer is distinct from its previously reported role in DNA damage, DNA replication, and DNA repair. We found that TK1 can promote LUAD tumor growth and metastasis in a non-canonical manner by activating Rho GTPase activity and growth and differentiation factor 15 (GDF15) expression. Taken together, our data suggest that TK1 may represent a potential target for development of LUAD therapy, due to its critical role in maintaining lung tumor growth and metastasis.

## Introduction

Lung cancer is the leading cause of cancer-related deaths in both men and women. Non-small cell lung cancer (NSCLC) accounts for ~80% of lung cancers, with lung adenocarcinoma (LUAD) constituting the most common type of NSCLC [1–3]. The severity of this disease and the limitations of current therapies, including immunotherapies, are highlighted by the fact that the five-year survival rate for LUAD patients with stage IIIB and stage IV disease is only 5% and 1%, respectively [1–3]. Therefore, an enhanced understanding of LUAD pathogenesis is needed to improve available therapies and provide meaningful clinical benefits to LUAD patients.

Cancer cells differ from normal cells in many different respects, and these features are collectively referred to as the hallmarks of cancer [4]. In particular, the specific metabolic needs of cancer cells have emerged as important cancer cell hallmarks [5–8]. Several studies have uncovered the importance of lung cancer-associated metabolic alterations and described their critical roles in lung cancer biology and therapy [9–11]. A previous study that analyzed KRAS/LKB1 dual-mutant (KL) NSCLC showed that human KL cells and tumors depend upon carbamoyl phosphate synthetase-1 (CPS-1) for survival [12]. Similarly, other metabolic enzymes, such as pyruvate carboxylase (PC), and metabolic pathways, such as the glutamine pathway and the *de novo* lipogenesis pathway, have been shown to be important for NSCLC cell survival [13–15].

However, new metabolic requirements for LUAD continue to be discovered, indicating that our understanding of the metabolic alterations in lung cancer, and the ways in which these cells utilize different metabolic pathways to promote tumor growth and evade responses to targeted therapeutic agents, remains incomplete.

Thymidine kinase 1 (TK1) is a cytosolic enzyme involved in pyrimidine metabolism that catalyzes the addition of a gamma-phosphate group to thymidine. TK1 is overexpressed in a number of different cancer types, and high levels of TK1 protein have been used as a biomarker for diagnosing and categorizing many types of cancers, including lung cancer [16–19]. Additionally, dual staining for TK1/CD31 was able to more accurately identify tumor vessels in colorectal carcinoma than staining for other markers, suggesting that TK1/CD31 dual staining may be a useful predictor of tumor responses to anti-angiogenic therapy [20]. However, the precise role that TK1 plays in LUAD and other cancer types, as well as its mechanisms-of-action, are still not fully understood.

Here, we investigated the role of TK1 in LUAD and found that this protein is overexpressed in LUAD patient-derived tissue, with higher expression levels associated with poor prognosis in LUAD patients. We further show that knockdown of TK1 inhibits tumor growth and metastatic attributes by inhibiting Rho GTPase activity and by reducing the expression of growth and differentiation factor 15 (GDF15). Collectively, our data identify TK1 as a key regulator of LUAD tumor growth and metastasis, and suggest that this protein may be utilized both as a predictive biomarker for poor prognosis in LUAD and as a target for LUAD therapy.

## Results

### TK1 is overexpressed in LUAD and its overexpression is associated with poor prognosis and cancer recurrence

**Fig 1:**
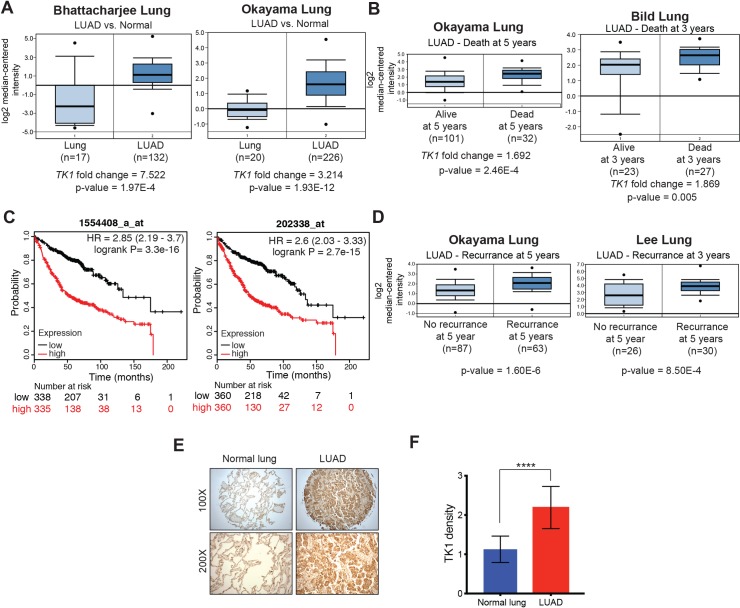
TK1 is upregulated in lung adenocarcinoma and predicts poor prognosis. **(A)** Lung adenocarcinoma (LUAD) datasets were analyzed for *TK1* mRNA expression; average fold change in *TK1* expression in patient-derived LUAD samples relative to normal lung tissues is shown. **(B)** Significant differences in *TK1* mRNA expression for patient-derived LUAD samples from patients who were alive or dead at 5 or 3 years; *P*-values for the indicated comparisons are shown. **(C)** Kaplan-Meier survival curves showing overall survival for LUAD patients with low (black) or high (red) *TK1*-expressing LUAD. **(D)** Comparison of *TK1* expression in patient-derived LUAD samples from subjects with no recurrence or recurrence at 5 years. *P*-value for the indicated comparison is shown. **(E)** Analysis of TK1 protein expression in a tissue microarray (TMA) containing LUAD and matched normal lung samples (n = 47 each). Immunohistochemical staining for TK1 in LUAD and matched normal lung tissue samples at 100× and 200× magnification; representative images are shown. **(F)** Analysis of immunohistochemical data from TMA with LUAD and matched normal lung tissue samples. The average densities of TK1 staining in LUAD and matched normal lung tissue are plotted and presented as the mean ± standard error of the mean (SEM); **** represents *P* < 0.0001.

While analyzing gene expression data from LUAD patient samples, we discovered significant upregulation of *TK1* mRNA in a large majority of LUAD patient samples, as compared to normal lung samples (Fig 1A, S1A Fig) [21–26]. In addition, LUAD patients with higher expression of *TK1* showed poor prognosis and reduced overall survival relative to those with lower *TK1* expression (Fig 1B and 1C and S1B Fig) [22, 27–29]. These patients with higher *TK1* mRNA levels also showed significantly higher incidence of disease recurrence (Fig 1D) [22, 30]. To further determine the significance of the enhanced *TK1* expression observed in LUAD mRNA expression datasets, we analyzed TK1 protein expression using immunohistochemistry in a tissue microarray (TMA) comprised of LUAD samples (n = 47) and matched normal lung tissues (n = 47). First, we validated the specificity of the TK1 antibody in separate immunoblot and immunofluorescence experiments (S1C and S1D Fig). In the TMA, we detected significantly higher TK1 protein expression in a large majority of patient-derived LUAD tumors, as compared to normal matched lung tissues (Fig 1E and 1F and S1 Table). Collectively, these results reveal that TK1 is overexpressed in LUAD, and this elevated expression is associated with poor prognosis and disease recurrence.

### TK1 expression is necessary for LUAD tumor growth and maintenance of metastatic attributes

**Fig 2:**
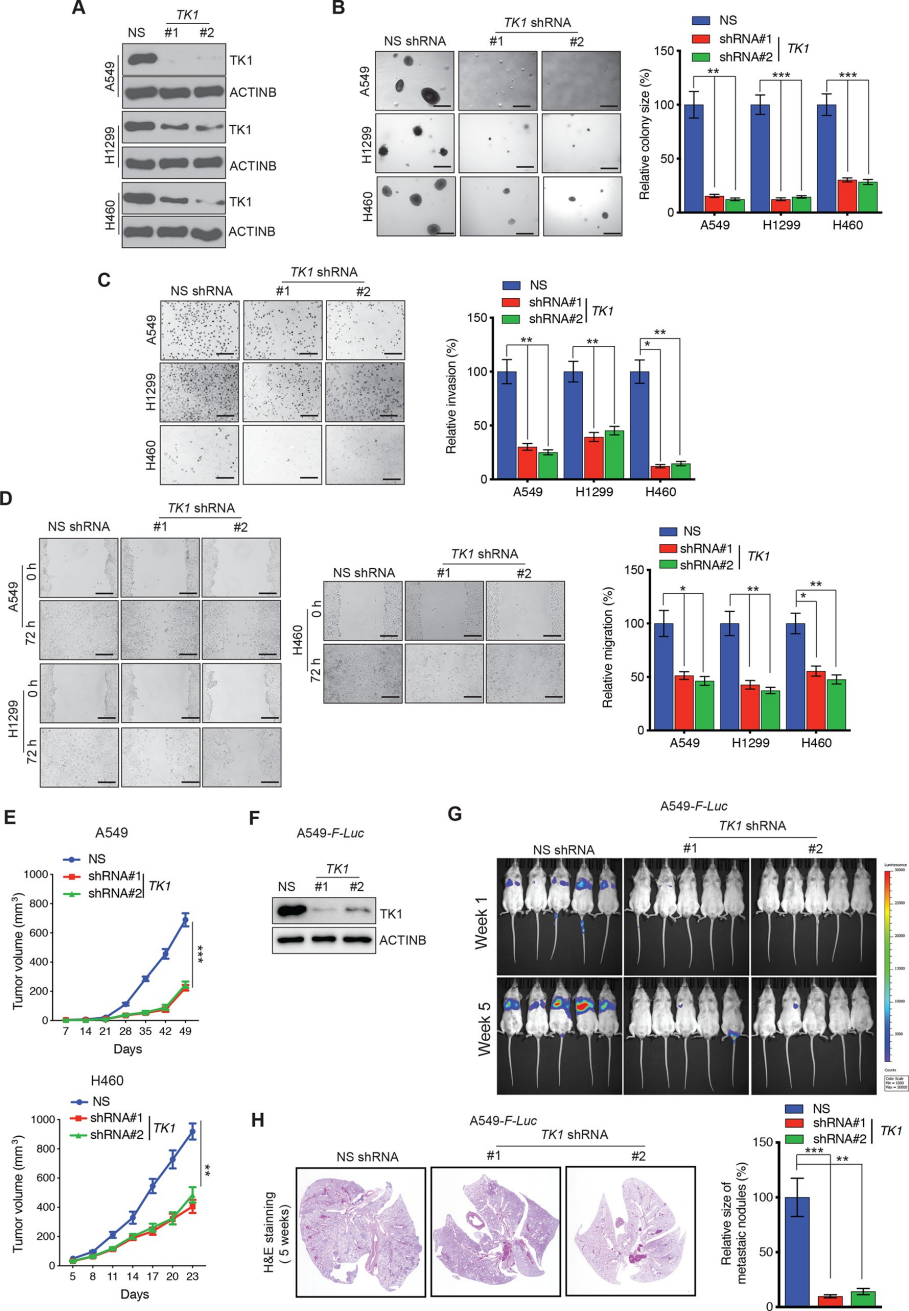
TK1 inhibition reduces growth and metastasis of LUAD cell lines. **(A)** TK1 levels were measured by immunoblot analysis in LUAD cell lines expressing *TK1* shRNAs or control, NS shRNA. ACTINB was used as a loading control. **(B)** (Left) Anchorage-independent growth was measured by soft-agar assay in LUAD cell lines expressing either *TK1* short hairpin RNA (shRNA) or a non-specific (NS) shRNA control. Representative images of soft-agar colonies from the indicated LUAD cell lines are shown. Scale bar, 500 μm. (Right) Plot showing relative colony sizes from the soft-agar assay presented in panel on left. **(C)** (Left) Matrigel invasion assays with the indicated LUAD cell lines expressing *TK1* shRNA or NS shRNA; representative images are shown. Scale bar, 200 μm. (Right) Relative invasion (%) from Matrigel assays shown in the left panel. **(D)** (Left) Wound-healing assays with LUAD cells expressing *TK1* shRNA or NS shRNA control. Representative images at the indicated times are shown. Scale bar, 200 μm. (Right) Relative migration (%) calculated from the data presented on the left. **(E)** LUAD cell lines expressing either *TK1* shRNA or NS shRNA were subcutaneously injected into the flanks of athymic nude mice (n = 3). Average tumor volumes at the indicated time points are shown. **(F)** TK1 levels were measured by immunoblot analysis in A549-*F-Luc* cells expressing *TK1* shRNAs or NS shRNA. ACTINB was used as a loading control. **(G)** A549-*F-Luc* cells expressing *TK1* shRNA or NS shRNA were administered to NSG mice (n = 5) *via* tail vein injection. Bioluminescence images of mice from the indicated groups at weeks 1 and 5 are shown. **(H)** (Left) Representative images of hematoxylin and eosin (H&E)-stained lung sections from the week 5 groups shown in panel G. (Right) Relative size of metastatic nodules in week 5 lungs with A549-*F-Luc* cells expressing *TK1* shRNA or NS shRNA. Data are presented as the mean ± SEM; *, **, and *** represent *P* < 0.05, *P* < 0.01, and *P* < 0.001, respectively.

The observation that TK1 overexpression in LUAD is predictive of tumor aggressiveness, as evidenced by its significant association with poor prognosis and LUAD recurrence (Fig 1C and 1D), led us to ask whether TK1 is important for LUAD tumor growth and metastasis. To this end, we first tested the effect of TK1 knockdown on LUAD tumor growth, using two sequence-independent short hairpin RNAs (shRNAs) to target TK1 in three different LUAD cell lines (A549, H1299 and H460) (Fig 2A and S2A Fig). Knockdown-validated LUAD cell lines were then tested for their ability to form colonies in soft-agar assays, as the measurement of anchorage-independent growth in soft-agar can serve as a surrogate assay for *in vivo* tumorigenesis [31, 32]. We found that TK1 knockdown in LUAD cells results in significantly reduced ability to form colonies in soft-agar (Fig 2B). We obtained similar results in the clonogenic assay (S2B Fig).

We then determined whether *TK1* knockdown can modulate the metastatic attributes of LUAD cells *in vitro* using Matrigel invasion and wound-healing migration assays. Our results demonstrate that *TK1* knockdown in LUAD cells leads to reduced invasion (Fig 2C) and reduced migration (Fig 2D), relative to cells transfected with control shRNA. The effect of TK1 knockdown on LUAD cell invasion was independent of the effect on proliferation, because in the timeframe in which invasion was analyzed, we did not see a significant effect of TK1 knockdown on LUAD cell proliferation (S2C Fig).

Based on these results, we next asked if TK1 knockdown inhibits LUAD tumor growth and metastasis *in vivo*. We first injected LUAD cells (A549 and H460) expressing either *TK1-*specific shRNAs or control, non-specific (NS) shRNA subcutaneously into the flanks of immunocompromised mice. Consistent with the results of the cell culture experiments, *TK1* knockdown led to significant inhibition of tumor growth *in vivo* for all the LUAD cell lines tested (Fig 2E). We also investigated the effect of *TK1* knockdown on the growth of metastatic lung tumors *in vivo*. Metastatic spread of LUAD to the other unaffected lung is one of most common forms of metastasis [33]. Therefore, to mimic that phenomenon, we injected firefly luciferase gene-labeled A549 cells (A549-*F-Luc*) expressing *TK1* shRNA or control NS shRNA into the tail veins of immunocompromised mice (Fig 2F). We found that *TK1* knockdown in A549 cells results in significantly reduced metastatic growth in lungs, as compared to cells expressing NS shRNAs (Fig 2G and 2H). Collectively, these results demonstrate that inhibition of TK1 blocks tumor growth and metastatic attributes of LUAD cells, both in cell culture and in mice.

### TK1 is transcriptionally upregulated by the transcription factor, MAZ

**Fig 3:**
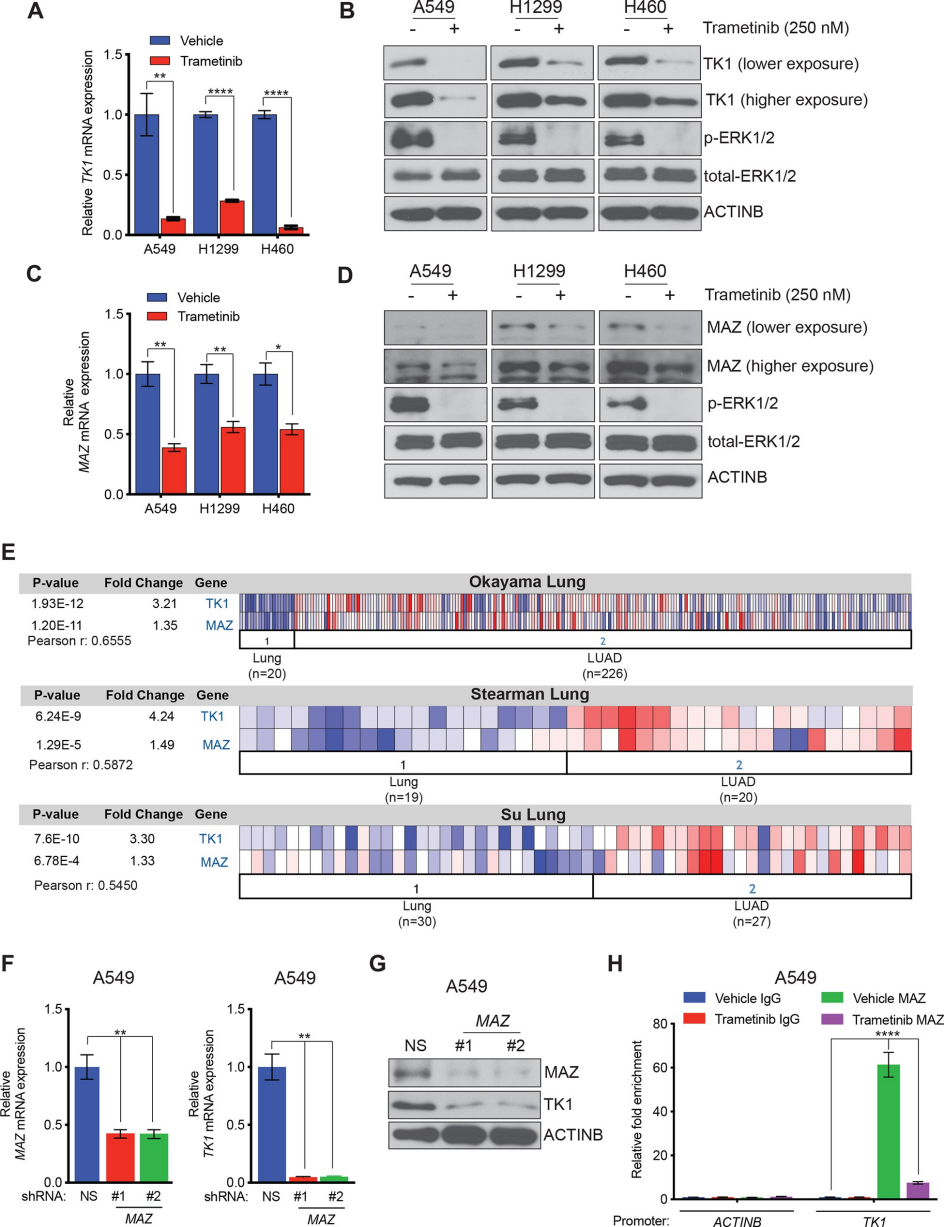
TK1 is transcriptionally upregulated by the transcription factor, MAZ. **(A)** *TK1* mRNA expression was measured by quantitative reverse transcriptase-PCR (qRT-PCR) in the indicated LUAD cell lines treated with dimethyl sulfoxide (DMSO) (-) or trametinib (250 nM) for 24 h. *TK1* mRNA expression in response to trametinib is plotted relative to treatment with DMSO. **(B)** LUAD cell lines were treated with DMSO (-) or trametinib (250 nM) for 24 h, and expression of the indicated proteins was measured by immunoblot analysis. ACTINB was used as a loading control. **(C)** LUAD cell lines were treated with DMSO (-) or trametinib (250 nM) for 24 h, and mRNA expression of the indicated genes was measured by qRT-PCR. Expression in response to trametinib is plotted relative to treatment with DMSO. **(D)** LUAD cell lines were treated with DMSO (-) or trametinib (250 nM) for 24 h, and expression of the indicated proteins was measured by immunoblot analysis. ACTINB was used as a loading control. **(E)** LUAD sample datasets were analyzed for *TK1* and *MAZ* mRNA expression using the Oncomine database; relative expression in each dataset is presented. **(F)** *MAZ* and *TK1* mRNA expression were measured by qRT-PCR in A549 cells expressing either *MAZ* shRNA or NS shRNA control; mRNA expression in *MAZ* shRNA-expressing cells is plotted relative expression in NS shRNA-expressing cells. **(G)** TK1 and MAZ protein levels were measured by immunoblot analysis in A549 cells expressing either *MAZ* shRNAs or NS shRNA control. ACTINB was used as a loading control. **(H)** MAZ recruitment to either the *TK1* promoter or the *ACTINB* promoter as a control was measured by chromatin immunoprecipitation (ChIP) assay in A549 cells that were treated with DMSO or trametinib (250 nM) for 24 h. IgG was used as a negative control for IP, and fold-enrichment relative to IgG is shown. The coordinates of MAZ-binding sites on the *TK1* promoter are shown in the top panel. Data are presented as the mean ± SEM; *, **, and **** represent *P* < 0.05, *P* < 0.01, and *P* < 0.0001, respectively.

Our results showed that TK1 was overexpressed at the mRNA level in LUAD, and previous reports have shown that the MAP kinase pathway is commonly activated in LUAD cells [34]. We therefore asked whether the MAP kinase pathway is necessary for TK1 transcriptional upregulation. LUAD cell lines (A549, H1299, and H460) were treated with the MEK inhibitor trametinib, or dimethyl sulfoxide (DMSO) control, and expression of TK1 was measured by quantitative reverse transcriptase-PCR (qRT-PCR) and immunoblot analysis. We found that treatment with trametinib results in the downregulation of both *TK1* mRNA (Fig 3A**)** and protein (Fig 3B), indicating that the MAP kinase pathway is necessary for transcriptional upregulation of *TK1*.

In order to identify candidate transcription factors that may be involved in MAP kinase pathway-dependent upregulation of TK1, we analyzed the promoter sequence of *TK1* using the rVista 2.0 and PROMO 3.0 programs [35, 36] and identified putative DNA binding sites for 38 transcription factors (S2 Table). We then determined which of the 38 candidate transcription factors identified in our analysis are regulated by the MAP kinase pathway. To this end, we treated three LUAD cell lines (A549, H1299, and H460) with trametinib or DMSO control and measured expression of each transcription factor using qRT-PCR and immunoblot analysis. We found that out of 38 transcription factors, only MAZ showed downregulation in response to trametinib treatment in all LUAD cell lines tested (Fig 3C and 3D and S3 Fig). In addition, analysis of the LUAD gene expression datasets revealed that TK1 overexpression is significantly correlated with elevated MAZ expression of LUAD patient samples (Fig 3E and S4 Fig**)** [22, 25, 26]. These results indicate that MAZ could play a role in the transcriptional regulation of TK1 in LUAD.

To further elucidate the role of MAZ in TK1 transcriptional regulation, we knocked down the expression of *MAZ* in A549 cells using shRNA and measured the expression of TK1 using qRT-PCR and immunoblot analysis (Fig 3F and 3G). Our data reveal that knockdown of *MAZ* results in significantly reduced levels of both *TK1* mRNA and TK1 protein (Fig 3F and 3G). We then performed chromatin immunoprecipitation (ChIP) assays to assess the recruitment of MAZ on the *TK1* promoter and determine whether *TK1* is a direct transcriptional target for MAZ. Our ChIP data revealed that MAZ binds to the *TK1* promoter, and this binding is inhibited by trametinib-mediated MAP kinase pathway inhibition (Fig 3H). Collectively, these results demonstrate that the transcription factor, MAZ, is involved in the transcriptional upregulation of TK1 in LUAD cells.

### TK1 knockdown induces increased DNA damage, independent of its ability to promote LUAD growth and metastasis

Previous studies have reported an important role for TK1 in DNA replication, DNA repair, and DNA damage control [37–39]. Therefore, we tested whether loss of TK1 results in increased DNA damage in LUAD cells by performing immunofluorescence staining for phosphorylated γH2AX, as increased γH2AX foci formation (γH2AX phosphorylation) is a marker for DNA damage [40]. We found that shRNA-mediated knockdown of TK1 in A549, H1299, and H460 cells results in increased γH2AX foci formation (γH2AX phosphorylation), relative to controls (S5A Fig).

We then asked whether knockdown of other nucleotide kinases (such as deoxycytidine kinase, DCK) results in increased DNA damage and determined if this knockdown affects LUAD tumor forming ability *in vitro*. To this end, we knocked down *DCK* using shRNAs (S5B and S5C Fig), and measured γH2AX phosphorylation, as well as the ability of knockdown cells to form colonies in soft-agar. Similar to *TK1* knockdown, *DCK* knockdown resulted in increased γH2AX phosphorylation (S5D Fig). However, unlike *TK1* knockdown, *DCK* knockdown in LUAD cells did not affect soft-agar growth, invasion, or migration (S5E–S5G Fig). In addition, *DCK* mRNA was not found to be upregulated in patient-derived LUAD samples when compared to normal lung samples (S6 Fig). Thus, another nucleotide kinase, DCK that similar to TK1 induces DNA damage when knocked down in LUAD cells does not influence the growth or metastatic attributes of LUAD cells. This suggests that the ability of TK1 to promote tumor growth and metastatic attributes of LUAD cells occurs independently of its role in the regulation of DNA damage.

### TK1 knockdown results in the inhibition of Rho GTPase activity and a decrease in the GTP/GDP ratio *via* reduced activation of ribonucleotide reductase

Based on the results above, we hypothesized that dTTP, the product of TK1, promotes cancer growth independent of role for TK1 as a regulator of DNA synthesis and repair. It has been shown that dTTP acts as an allosteric activator of ribonucleotide reductase (RNR) [41, 42], which preferentially generates dGDP from GDP. This phylogenetically conserved regulatory mechanism helps to maintain physiologically stable ratios of *de novo* synthesized dNTP pools [43, 44]. The altered GTP/GDP ratio that results from dTTP-induced dGDP synthesis leads to a depletion of GDP, which can affect the activities of several G-coupled proteins, including Ras and RhoA [45].

**Fig 4:**
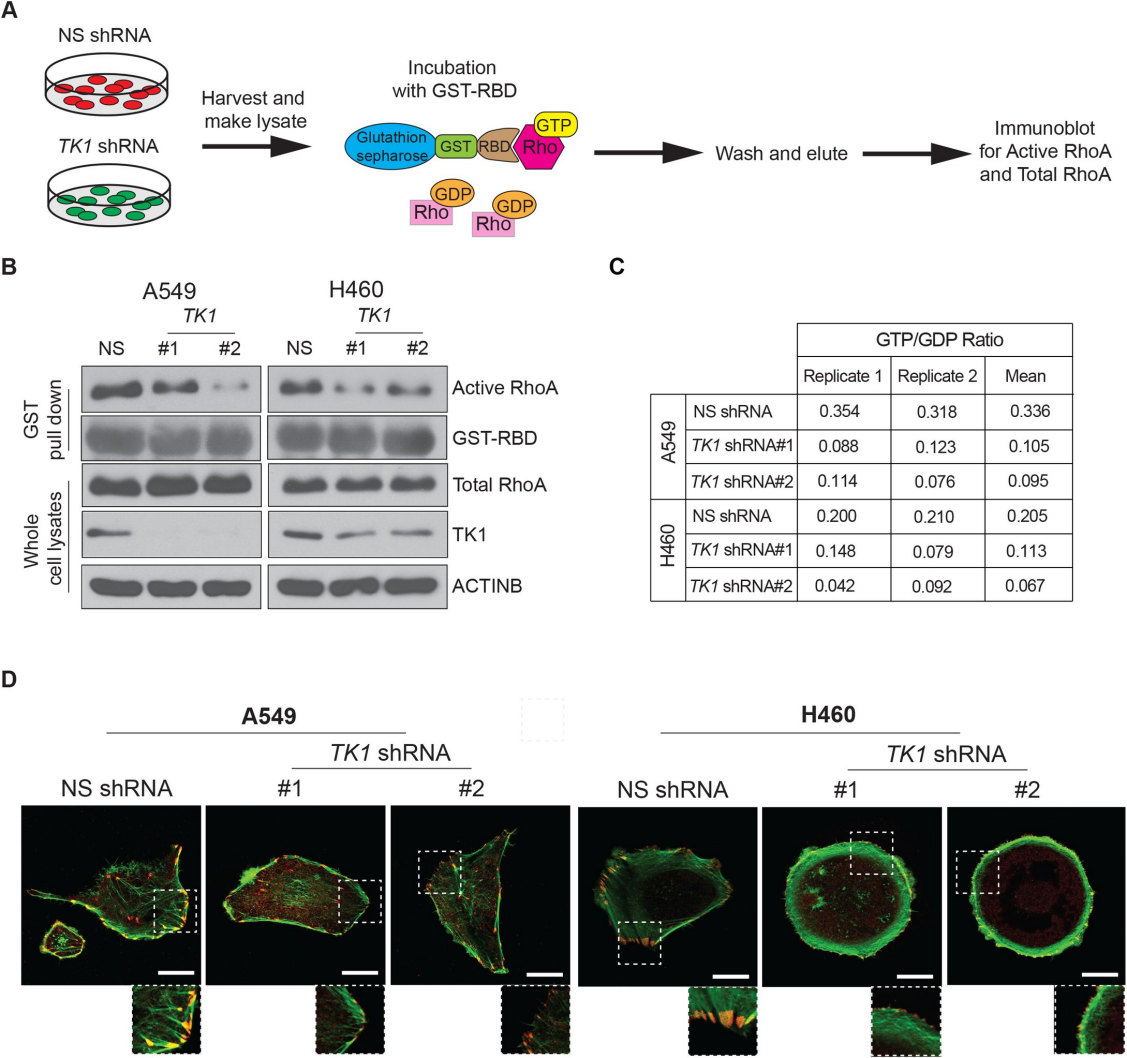
TK1 knockdown affects Rho GTPase activity by reducing the activation of ribonucleotide reductase in LUAD cells. **(A)** Experimental design for the glutathione S-transferase (GST) pull-down assay used to analyze active RhoA. **(B)** Active RhoA was measured in the indicated LUAD cell lines expressing *TK1* shRNA or NS shRNA control using GST pull-down assays and immunoblot analysis. GST-RBD was used as a control in the pull-down assay, and total RhoA in whole-cell lysates was used as a loading control for immunoblot analysis. **(C)** GTP/GDP ratios were measured in the indicated LUAD cell lines expressing *TK1* shRNA or NS shRNA control using high-performance liquid chromatography-tandem mass spectrometry (HPLC-MS/MS). **(D)** Active RhoA was measured in the indicated LUAD cell lines expressing *TK1* shRNA or NS shRNA using actin (green)/vinculin (red) immunofluorescence and confocal microscopy. Representative images are shown. Scale bar, 20 μm.

Rho GTPases are evolutionarily conserved small GTPases that have been shown to promote cancer growth and progression *via* regulation of actin cytoskeleton, cell-cell signaling, and other mechanisms [46–48]. Therefore, we hypothesized that TK1 knockdown-mediated reduction in cancer growth and progression results from the deregulation of Rho GTPase activity through an altered GTP/GDP ratio. To test this possibility, we first measured RhoA activation status in LUAD cell lines expressing *TK1* shRNAs (Fig 4A). Indeed, we observed reduced activation of RhoA after *TK1* knockdown (Fig 4B), which correlates with a reduced GTP/GDP ratio in *TK1* knockdown cells (Fig 4C). A mark of reduced RhoA activity is a decrease in actin stress fibers. Therefore, we also measured actin stress fibers in cells expressing TK1 shRNAs and found that TK1 knockdown leads to a significant reduction in actin stress fibers (Fig 4D), which further supports a model whereby TK1 loss leads to reduced RhoA activation.

To confirm whether TK1 activity and dTTP are important for RhoA activation, we knocked down the expression of other enzymes in the dTTP synthesis pathway in LUAD cells, and measured RhoA activation (S7A and S7B Fig). Similar to *TK1* knockdown, knockdown of deoxythymidylate kinase (*DTYMK)* and nucleoside diphosphate kinase 1 (*NME1)* inhibits the anchorage-independent growth of A549 cells (S7C Fig), and this is correlated with a reduced activation of RhoA in these cells (S7D and S7E Fig). Collectively, these results show that TK1 induces RhoA activation through an altered GTP/GDP ratio, which is needed for the growth promoting activity of TK1.

### TK1 promotes LUAD growth and metastatic attributes through induction of GDF15 expression

**Fig 5:**
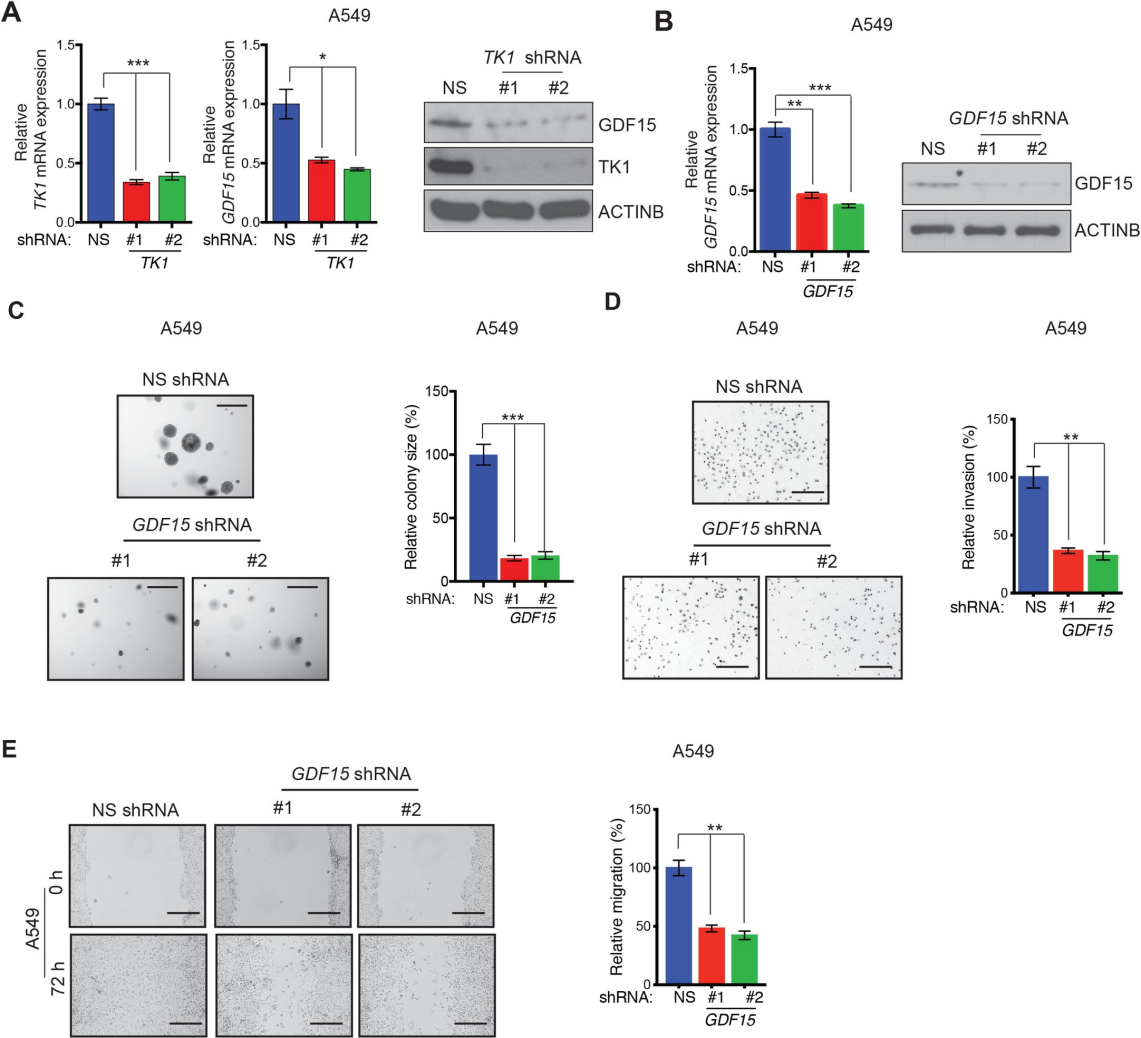
*TK1* knockdown inhibits GDF15 expression, and GDF15 is necessary for growth and metastatic attributes in LUAD cells. **(A)** (Left) *GDF15* mRNA expression was measured by qRT-PCR in A549 cells expressing either *TK1* shRNA or NS shRNA control. *GDF15* mRNA levels in *TK1* shRNA-expressing cells are plotted relative to NS shRNA-expressing cells. (Right) Expression of the indicated proteins was measured by immunoblot analysis in A549 cells expressing either *TK1* shRNA or NS shRNA control. ACTINB was used as a loading control. **(B)** (Left) *GDF15* mRNA expression was measured by qRT-PCR in A549 cells expressing either *GDF15* shRNA or NS shRNA control. *GDF15* mRNA levels in *GDF15* shRNA-expressing cells are plotted relative to NS shRNA-expressing cells. (Right) Expression of the indicated proteins was measured by immunoblot analysis in A549 cells expressing either *GDF15* shRNA or NS shRNA control. ACTINB was used as a loading control. **(C) (**Left) Anchorage-independent growth was measured by soft-agar assay in A549 cell lines expressing either *GDF15* shRNA or a NS shRNA control. Representative images of soft-agar colonies from A549 cells expressing either *GDF15* shRNA or NS shRNA are shown. Scale bar, 500 μm. (Right) Plot showing relative colony sizes from the soft-agar assay shown on the left. **(D**) (Left) Matrigel invasion assays with the A549 cells expressing either *GDF15* shRNA or NS shRNA control; representative images are shown. Scale bar, 200 μm. (Right) Relative invasion (%) from the Matrigel assays shown in panel shown on left. **(E)** (Left) Wound-healing assays with LUAD cells expressing *GDF15* shRNA or NS shRNA control. Representative images at the indicated times are shown. Scale bar, 200 μm. (Right) Relative migration (%) from the data shown on the left. Data are presented as the mean ± SEM; *, **, and *** represent *P* < 0.05, *P* < 0.01, and *P* < 0.001, respectively.

To further elucidate the mechanism of TK1 activity, we performed gene expression analysis of A549 cells expressing *TK1* shRNAs and the NS shRNA control using the Illumina BeadChip array platform. Analysis of gene expression data revealed that five genes––growth and differentiation factor 15 (*GDF15)*, high mobility group box 3 (*HMGB3*), monocyte to macrophage differentiation associated (*MMD*), homeodomain interacting protein kinase 2 (*HIPK2*) and hypoxia inducible lipid droplet associated (*HILPDA*)—are significantly downregulated in *TK1* knockdown cells (Fig 5A, S8A Fig and S3 Table).

To determine if any of these genes acts as a downstream mediator of TK1 function, we first knocked down the expression of all five genes individually using shRNA in A549 cells (Fig 5B and S8B Fig) and measured the ability of knockdown cells to form colonies in soft-agar. We found that out of five candidates tested, only GDF15 knockdown, similar to *TK1*, results in reduced colony formation in a soft-agar assay (Fig 5C and S8C Fig). We then determined whether GDF15 knockdown affects the metastatic attributes of LUAD cells by performing Matrigel invasion and wound-healing migration assays with A549 cells expressing GDF15 shRNAs. We found that, similarly to *TK1* knockdown, *GDF15* knockdown inhibited the ability of A549 cells to invade (Fig 5D) and migrate (Fig 5E). However, that effect was independent of the ability of GDF15 to regulate RhoA activity because GDF15 knockdown did not result in reduced RhoA activity (S8D Fig).

**Fig 6:**
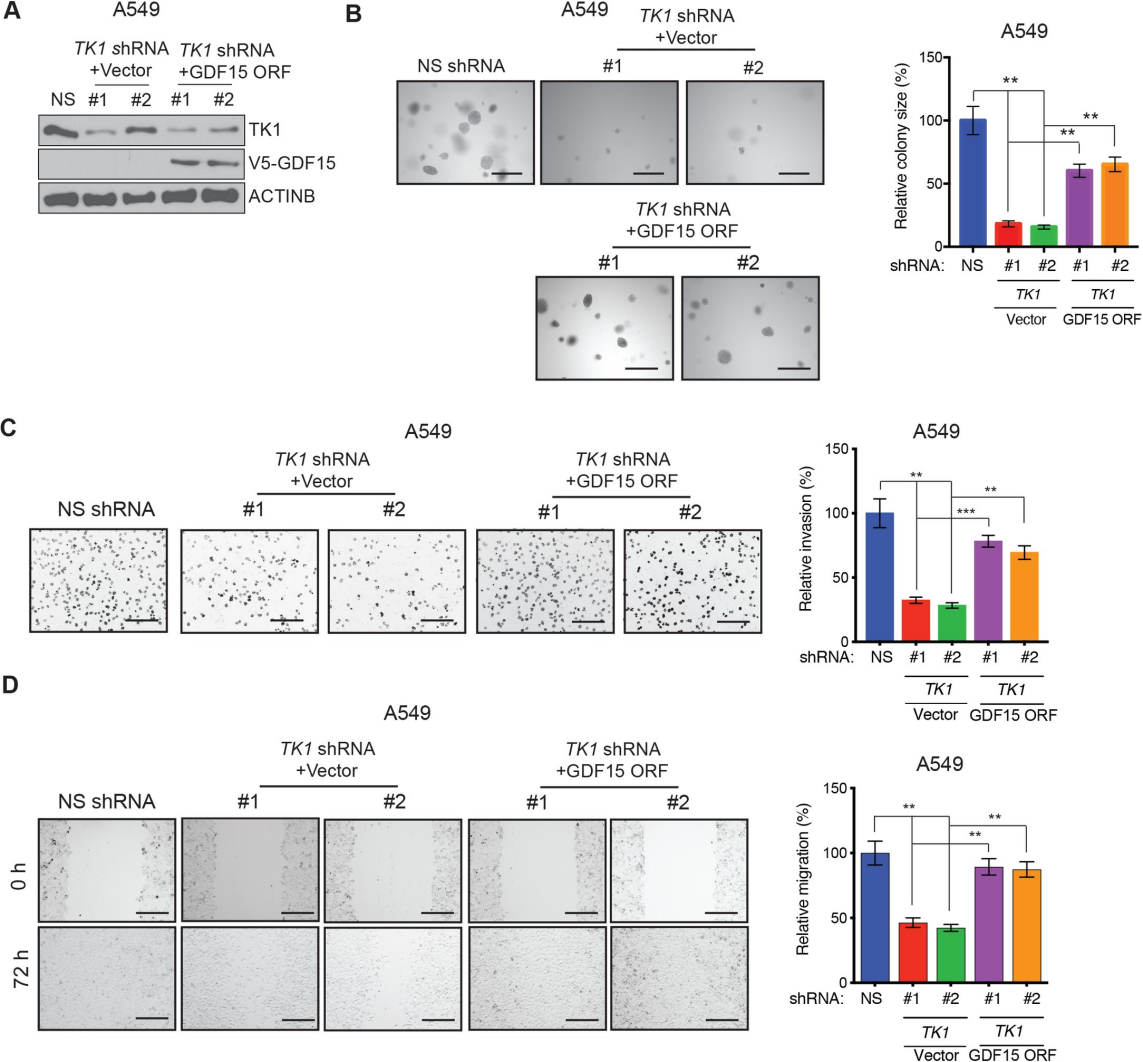
Ectopic expression of GDF15 can partially rescue *TK1* knockdown-induced inhibition of LUAD growth and metastatic attributes. **(A)** Expression of the indicated proteins was measured by immunoblot analysis in A549 cells expressing *TK1* shRNA or NS shRNA control in combination with the *GDF15* expression vector or empty vector (pLX304) control. ACTINB was used as a loading control. **(B)** (Left) Anchorage-independent growth was measured by soft-agar assay in A549 cells expressing *TK1* shRNA or NS shRNA control in combination with the *GDF15* expression vector or empty vector (pLX304) control. Representative images of soft-agar colonies for the indicated conditions are shown. Scale bar, 500 μm. (Right) Plot showing relative colony sizes from the soft-agar assay shown on the left. **(C)** (Left) Matrigel invasion assays with A549 cells expressing *TK1* shRNA or NS shRNA control in combination with the *GDF15* expression vector or empty vector (pLX304) control; representative images are shown. Scale bar, 200 μm. (Right) Relative invasion (%) from the Matrigel invasion assays shown on the left. **(D)** Wound-healing assays with A549 cells expressing *TK1* shRNA or NS shRNA in combination with the *GDF15* expression vector or empty vector (pLX304) control. Representative images at the indicated times are shown. Scale bar, 200 μm. (Right) Relative migration (%) calculated from the data shown on the left. Data are presented as the mean ± SEM; ** and *** represent *P* < 0.01 and *P <* 0.001, respectively.

Finally, to directly test whether GDF15 acts downstream of TK1, we determined if ectopic expression of GDF15 can rescue the TK1 loss-induced inhibition of LUAD growth and metastatic attributes. To this end, we ectopically expressed GDF15 in A549 cells expressing *TK1* shRNA (Fig 6A) and performed soft-agar, Matrigel invasion, and wound-healing assays. We found that ectopic expression of GDF15 can partially rescue the growth of A549 cells knocked down for *TK1* expression in the soft-agar assay (Fig 6B). In addition, ectopic GDF15 expression was able to restore the invasiveness (Fig 6C) and the migration properties (Fig 6D) of LUAD cells. Collectively, these results demonstrate that TK1 promotes LUAD tumor growth, in part, by stimulating the expression of GDF15.

## Discussion

**Fig 7:**
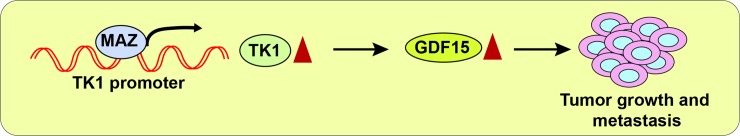
Model showing the mechanism by which TK1 facilitates LUAD tumor growth and metastasis. We find that TK1 promotes the expression of GDF15, which acts downstream of TK1 to mediate its ability to promote tumor growth and metastatic attributes in LUAD cells.

Lung adenocarcinoma, the most common type of lung cancer, remains a clinical challenge even with significant developments in the field of targeted therapeutics and immunotherapies. This highlights the need for an enhanced understanding of LUAD with the goal of developing better treatment options and improved management strategies for this disease. The metabolic evolution of cancer, including that of lung cancer, affects almost all aspects of this disease, including tumor initiation, disease progression, and response to therapies. In this study, we identified TK1 as a metabolic enzyme that is overexpressed in LUAD and promotes LUAD tumor and metastatic growth. The results of our findings and our proposed model by which TK1 functions in LUAD are presented in Fig 7 and summarized below.

### TK1 overexpression in LUAD and its association with poor prognosis

*TK1* encodes a cytosolic enzyme that adds a gamma-phosphate group to thymidine to generate dTMP. This is the first step in the biosynthesis of dTTP, one of the key components required for DNA replication. TK1 is overexpressed in a number of different cancers [16–18], and several studies have used expression of this protein as a biomarker for cancer detection. A previous study, showed that serum TK1 is a potential biomarker for early cancer detection in people at risk for developing, or those who already have, precancerous growth [16]. Here, we found that *TK1* mRNA is overexpressed in patient-derived LUAD samples, as compared to normal tissue. Our mechanistic studies further revealed that this occurs, in part, *via* the action of the transcription factor, MAZ, in a MAP kinase pathway-dependent manner. These data are consistent with a previous study, which showed that TK1 overexpression is associated with reduced overall survival in lung cancer patients [17]. Overall, these studies indicate that TK1 overexpression might be indicative of a more aggressive form of lung cancer in general, and this protein may have predictive value in LUAD, in particular.

### TK1 as a facilitator of LUAD tumor growth and metastasis

Not all genes that are upregulated in cancer and/or predict cancer survival necessarily act as drivers of cancer growth and progression. Therefore, functional validation is required to definitively establish a role in driving tumor growth. However, even with abundant evidence for the overexpression of TK1 in a wide variety of cancer and the association of this protein with poor prognosis, no study thus far has analyzed the functional implication of TK1 inhibition on tumor growth and progression. We knocked down *TK1* expression using shRNA and performed a series of cell culture and mouse-based studies to assess the effect on knockdown on tumor growth and metastasis. Our results show that knockdown of TK1 significantly inhibits tumor and metastatic growth, both in cell culture and in mice, suggesting that TK1 expression is required for tumor growth and the metastatic attributes of LUAD cells. We recognize that because TK1 affects cell proliferation, the ability to affect cell proliferation may to some extent contribute to the ability of TK1 to promote other metastatic attributes (e.g., migration). Our results show, however, that in a time frame in which the loss of TK1 does not affect cell proliferation, that the loss does result in decrease in cancer cell invasiveness, indicating an effect on metastatic attributes that can be separated from the primary effect on cell proliferation. Furthermore, it is quite common to find deregulated cancer genes (e.g., tumor suppressors or oncogenes) that can promote both tumor growth/progression attributes and metastasis/metastatic attributes. For example, mutant p53 has been shown to promote both tumor progression and metastasis [49]. Similarly, oncogenic KRAS, which is a well-known oncogene necessary for cancer initiation and maintenance of tumor growth, can also drive invasion and maintain metastasis in colorectal cancer [50]. Several other examples in which cancer genes promote both tumor growth and metastasis have been described [51–53]. Taken together, these results provide an important validation for the proposed role of TK1 as a facilitator of LUAD tumor and metastatic growth. Notably, because the overexpression of TK1 and its association with poor prognosis has been detected in the clinical samples from a number of other cancer types, we expect that similar studies will establish the role of TK1 as a general driver for cancer growth and metastasis.

### A mechanism of TK1 action that is independent of DNA damage regulation

Previous studies have reported an important role for TK1 in DNA replication, DNA repair, and DNA damage control [37–39]. TK1 is required to generate and maintain the deoxyribonucleotide dTTP, which is needed for DNA replication and recovery from DNA damage [38], thereby preventing DNA damage-induced cell death. As expected, we found that TK1 knockdown results in increased DNA damage in LUAD cells, and a similar increase in DNA damage was observed in cells knocked down for the expression of another nucleotide kinase, DCK. However, knockdown of DCK failed to inhibit LUAD tumor growth, indicating that the DNA damage that results from TK1 knockdown is unlikely to play a role in the inhibition of LUAD tumor growth and metastasis.

Therefore, in order to elucidate the mechanism of action for TK1 in LUAD, we performed both a targeted and an unbiased gene expression analysis in TK1 knockdown cells. Rho GTPases promote cancer growth and progression *via* regulation of the actin cytoskeleton, cell-cell signaling, and other mechanisms [46–48]. Here, we hypothesized that the TK1 knockdown-mediated inhibition of cancer growth and progression results from decreased pools of dTTP, which leads to an altered GTP/GDP ratio and the deregulation of Rho GTPase activity. Consistent with this hypothesis, we found that knockdown of TK1 results in reduced RhoA activation and a decreased GTP/GDP ratio.

The Ras-like superfamily of small G-proteins includes both the Ras and Rho GTPase families, both of which contain proteins that are known to be deregulated in cancer [54]. Members of the Ras family of proteins have very high affinities for guanyl nucleotides (in the picomolar range) [55], and in addition, their affinity for GTP is higher than for GDP [56]. This indicates that small perturbations in the cellular GTP/GDP ratio would be unlikely to affect GTP loading or GDP dissociation for the Ras family of proteins. In contrast, members of the Rho family of proteins have affinities toward guanyl nucleotides that are many orders of magnitude lower than Ras family proteins (0.1–0.6 μM), making them susceptible to small changes in the GTP/GDP ratio [57]. As noted above, Rho GTPases have also been shown to be involved in promoting tumor growth and progression [58–62]. Therefore, our data demonstrating that reduced Rho GTPase activity that results from knockdown of TK1 can block LUAD tumor growth and metastasis are consistent with the published literature.

In addition, from our unbiased gene expression analysis, we identified GDF15 as a gene whose expression is downregulated as a result of TK1 loss. GDF15 is a member of the bone morphogenetic protein (BMP) subfamily of the transforming growth factor-beta (TGF-β) superfamily. GDF15 is a target of the tumor suppressor p53 [63] and can inhibit atherosclerosis by attenuating CCR2-mediated macrophage chemotaxis [64]. GDF15 has also been shown to protect transformed cells from macrophages, to promote tumor development *in vivo*, to regulate bone metastasis, and to induce LUAD cell proliferation [65–68]. In particular, higher levels of circulating GDF15 have been shown to be a biomarker of bone metastasis that can be combined with other biomarkers to more accurately predict incidences of bone metastasis [66].

Here, we found that GDF15 is an important mediator of TK1 function, as the TK1 knockdown-induced reduction in LUAD tumor growth and metastasis can be rescued by ectopic expression of GDF15. Thus, our findings indicate that the loss of GDF15 expression and reduced RhoA activity resulting from loss of TK1 *via* shRNA silencing leads to reduced tumor and metastatic activity in LUAD cells.

## Materials and methods

### Ethical statement

All animal experiments were approved by the Institutional Animal Care and Use Committee (IACUC) at Yale University and were performed in accordance with the IACUC guidelines.

### Cell culture

A549, H1299, and H460 cell lines were obtained from the American Type Culture Collection (ATCC, Manassas, VA, USA) and maintained as recommended by ATCC. A549 and H1299 cells were grown in Dulbecco's Modified Eagle Medium (DMEM; Life Technologies, Thermo Fisher Scientific, Waltham, MA, USA), supplemented with 10% fetal bovine serum (FBS; Life Technologies, Thermo Fisher Scientific) and 1% penicillin/streptomycin (Life Technologies), at a CO_2_ concentration of 5%. H460 cells were grown in Roswell Park Memorial Institute (RPMI)-1640 Medium (Life Technologies, Thermo Fisher Scientific), supplemented with 10% FBS and 1% penicillin/streptomycin, in 5% CO_2_.

### shRNAs, transfection, lentivirus preparation, and stable cell line generation

All shRNAs were obtained from Open Biosystems (Dharmacon, Lafayette, CO, USA) and are listed in S4 Table. Lentiviral particles expressing individual shRNAs were generated by co-transfecting shRNA plasmids with the lentiviral packaging plasmids, pSPAX2 and pMD2.G, into 293T cells, using Effectene Transfection Reagent (QIAGEN, Hilden, Germany), according to the manufacturer instructions. Viruses were filtered using a 0.45-μm sterile filter. Stable cell lines were generated by infecting various LUAD cell lines with shRNA lentivirus particles in 12-well plates, followed by selection with puromycin (0.5–0.75 μg/ml).

### RNA preparation, cDNA synthesis, and qRT-PCR analysis

Total RNA was extracted using TRIzol (Invitrogen, Thermo Fisher Scientific) and purified using the RNeasy Mini Kit (QIAGEN), according to the manufacturer’s instructions. cDNA was generated using the ProtoScript First Strand cDNA Synthesis Kit (New England Biolabs, Ipswich, MA, USA), and quantitative real-time PCR was performed using Power SYBR Green Master Mix (Life Technologies, Thermo Fisher Scientific). Oligonucleotide sequences used for qRT-PCR analyses are listed in S4 Table.

### Immunoblot analysis

Cells were washed with ice-cold phosphate-buffered saline (PBS) and lysed in ice-cold IP lysis buffer (Thermo Fisher Scientific), containing protease inhibitor (Roche, Basel, Switzerland) and phosphatase inhibitor cocktail (Sigma-Aldrich, St. Louis, MO, USA). Briefly, lysed samples were centrifuged at 12,000 rpm for 40 min, and clarified supernatants were stored at –80°C. Protein concentrations were determined using Bradford Protein Assay Reagent (Bio-Rad Laboratories, Hercules, CA, USA). Equal amounts of protein samples (50–100 μg) were electrophoresed on 6–12% sodium dodecyl sulfate (SDS)-polyacrylamide gels and transferred onto polyvinylidene difluoride (PVDF) membranes (Millipore, Burlington, MA, USA). Membranes were blocked and probed with primary antibodies. After washing, membranes were incubated with the appropriate horseradish peroxidase (HRP)-conjugated secondary antibodies (1:2,000) (GE Healthcare Life Sciences, Malborough, MA, USA), and blots were developed using SuperSignal West Pico or Femto Chemiluminescent Substrate (Thermo Fisher Scientific). All antibodies used for immunoblotting are listed in S4 Table.

### Soft-agar assays

LUAD cells (5 × 10^3^) stably expressing the indicated shRNA or cDNA constructs were seeded onto 0.4% low-melting-point agarose (Sigma-Aldrich), layered on top of 0.8% agarose. After 3–4 weeks of incubation, colonies were stained with a 0.005% crystal violet solution and imaged using an inverted light microscope (Olympus). Colony size was measured using microscopy and plotted as percent relative colony size when compared with control cells. Colony numbers were counted using ImageJ software (https://imagej.nih.gov/ij/). Statistical analysis was performed using the Student’s t-test in GraphPad Prism, version 7.0 (GraphPad Software, San Diego, California, USA; www.graphpad.com).

### Clonogenic assay

LAUD cells were plated in 6-well culture plates (5 × 10^3^ cells/well). The medium was changed every 3 days. After 10 days, the cells were stained with a 0.005% Coomassie Brilliant Blue R-250 solution (Bio-Rad, USA), and the plates were imaged using an Epson Perfection V850 Pro Photo Scanner (USA).

### MTT assay

LUAD cells were plated at a density of 5×10^3^ cells/well in 96-well plates. After 20 h, the medium was removed, 20 μl methylthiazole tetrazolium (MTT; 5 mg/ml in PBS; Sigma-Aldrich, MO, USA) was added, and the cells were incubated for another 2 h at 37°C. The resulting formazan crystals were solubilized in 100 μl DMSO, and absorbance was measured at 570 nm with a reference wavelength of 630 nm.

### Matrigel invasion assays

Invasion assays were performed in BioCoat Growth Factor Reduced Matrigel Invasion Chambers (Cat#354483, BD Biosciences, Franklin Lakes, NY, USA), using LUAD cells expressing the indicated shRNAs. Cells were serum-starved for 6 h, and then 5 × 10^4^ cells/insert were seeded in triplicate into the top chamber, containing low-serum medium (0.2% FBS). Cells were incubated for 20 h to allow invasion toward the serum-rich medium (10% FBS) in the bottom well. The number of cells invading the Matrigel was quantified by DAPI staining and imaging; 8–12 fields per membrane were counted, and nuclei quantification was performed using ImageJ software (https://imagej.nih.gov/ij/).

### Wound-healing assays

LUAD cells expressing the indicated shRNAs were seeded at a density of 2 × 10^5^ cells per well and grown in 12-well plates until fully confluent. A scratch was then created using a sterile 20-μl pipette tip, and cell migration into the wound was monitored at 0, 12, 24, and 72 h using light microscopy. Quantification of wound healing was performed using ImageJ software (https://imagej.nih.gov/ij/).

### Chromatin immunoprecipitation (ChIP) assays

The *TK1* promoter sequence was downloaded from the University of California, Santa Cruz (UCSC) genome browser and analyzed using PROMO 3.0 (http://alggen.lsi.upc.es/cgi-bin/promo_v3/promo/promoinit.cgi?dirDB=TF_8.3) and rVista 2.0 (https://rvista.dcode.org) software. ChIP experiments were performed as described previously [69]. Cell lysates were incubated with specific antibodies as required (listed in S4 Table), with the IgG antibody used as a control. Normalized Ct (ΔCt) values were calculated by subtracting the Ct of input DNA from that of immunoprecipitated DNA (ΔCt = Ct[IP] − Ct[input]). The relative fold-enrichment of a transcription factor at its target site was calculated using the formula 2^−(ΔCt(T)−ΔCt(Actb))^, where ΔCt(T) and ΔCt(Actb) are the ΔCt values of the target and *β*-*ACTIN* (negative control) primers, respectively.

### Bioinformatic analysis of lung adenocarcinoma datasets

LUAD datasets were downloaded from Oncomine (https://www.oncomine.org), analyzed for *TK1* expression, and graphed as box plots to compare LUAD samples with normal lung tissue. We analyzed survival, recurrence, and the LUAD stage in relation to TK1 expression. In the Bhattacharjee lung dataset [21], 139 LUAD, 21 squamous cell lung carcinoma, 20 lung carcinoid tumor, 6 small cell lung carcinoma, and 17 normal lung samples were analyzed on Affymetrix U95A microarrays. Sample data include type, age, M stage, maximum tumor percentage, N stage, primary/metastatic, recurrence, sex, site of metastasis, smoking rate (packs per year), stage, survival, and T stage. The Okayama lung dataset [22] includes 226 LUAD and 20 normal lung samples that were analyzed on the Human Genome U133 Plus 2.0 Array. Sample data include EGFR mutation, KRAS mutation, EML4-ALK gene fusion, stage, recurrence, survival status, and others. The Selamat Lung Dataset [23] contains 58 LUAD and 58 normal lung (57 paired) samples that were analyzed on the Illumina HumanWG-6 v3.0 Expression Beadchip Array. Sample data include age, race/ethnicity, smoking status, and stage, as well as KRAS, EGFR, and STK11 mutation status. The Garber lung dataset [24] includes 67 lung carcinoma samples of various types and six normal lung samples that were analyzed on cDNA microarrays. Sample data include type, grade, TNM stage, and survival. For the Stearman lung dataset [25], samples from 10 invasive non-small cell LUADs and 10 adjacent normal tissues were analyzed on Affymetrix HG-U95Av2 arrays. With one exception, arrays were run in duplicate, generating 39 analyzed samples. Nine of the 10 patients had a history of smoking. The Su lung dataset [26] contains 66 lung samples that were analyzed on Affymetrix U133A microarrays. Samples include 26 LUADs with paired adjacent normal controls, 1 large cell lung carcinoma with paired adjacent normal control, 2 tissue mixtures, 2 commercial human normal lung tissues, 1 normal lung cell line, and 7 lung cancer cell lines. The Hou lung dataset [28] includes 91 non-small cell lung carcinoma and 65 adjacent normal lung samples that were analyzed on the Human Genome U133 Plus 2.0 Array. Sample data include age, sex, cancer sample site, and survival. The Bild lung dataset [27] contains 111 non-small cell lung carcinoma samples that were analyzed on Affymetrix Human Genome U133 Plus 2.0 microarrays. Sample data include type, survival, Ras mutation, stage, age, and sex. The Lee lung dataset [30] analyzed 75 squamous cell lung carcinoma and 63 LUAD samples on the Human Genome U133 Plus 2.0 Array. Sample data include age, sex, grade, TN stage, stage, recurrence status, and others.

### Plasmids and cloning

The LentiORF-*GDF15* expression vector was obtained from GE Dharmacon (Accession: BC000529, Clone ID: ccsbBroad304_02182). The glutathione S-transferase (GST)-Rho-binding domain (RBD) plasmid was a gift from Martin Schwartz (Addgene plasmid # 15247) and has been described previously [70]. Construct details are provided in S4 Table.

### Microarray analysis and processing

Total RNA was isolated from cells grown in 100-mm culture dishes, as described above, and samples were cleaned-up using RNeasy Mini Spin Columns (QIAGEN). For microarray experiments, total RNA was isolated from A549 cells expressing either a control NS shRNA or one of two *TK1* shRNA sequences, and this was used to generate labeled antisense RNA. All antisense RNAs were produced using the Ambion MessageAmp Kit and hybridized to the Illumina HumanHT-12 V4.0 Expression BeadChip array (Illumina, San Diego, CA, USA). Microarray data were processed using GenomeStudio (Illumina), log2-transformed, and quantile-normalized using the lumi package of Bioconductor. All samples passed a quality-control assessment, which included checking various control plots, as suggested by Illumina, as well as other standard microarray-related analyses. Differential expression analyses were performed using the limma package, and a moderated t-test, with a Benjamini-Hochberg multiple testing correction procedure, was used to determine statistical significance (adjusted *P*-value < 0.05). Pathway analyses for differentially expressed genes from each comparison were performed using MetaCore (version 6.8 build 29806; GeneGo). All microarray data were submitted to the Gene Expression Omnibus (Accession number: GSE90483).

### Immunohistochemistry

Formalin-fixed, paraffin-embedded tissue microarray (TMA) slides, containing LUAD and matched normal lung tissues, were obtained from US Biomax, Inc. (Cat. No. LC100013a; Derwood, MD, USA). Briefly, following deparaffinization of the slides, antigen retrieval was performed in citrate buffer (pH 6.0) at 97°C for 20 min using the Lab Vision PT Module (Thermo Scientific). Endogenous peroxides were blocked using hydrogen peroxide for 30 min. The slides were then washed with 1× Tris-buffered saline (TBS), and proteins were blocked using 0.3% bovine serum albumin (BSA) for 30 min. Slides were incubated in TK1 antibody (dilution 1:500), followed by secondary anti-rabbit HRP-conjugated antibody (Dako, Jena, Germany). Slides were then stained using the Dako Liquid DAB+ Substrate Chromogen System (Dako) and counterstained with Dako Automation Hematoxylin Histological Staining Reagent (Dako). TK1 staining was scored by Dr. Guoping Cai, who was blinded regarding the identity of the samples. All antibodies used for immunohistochemistry analyses are listed in S4 Table.

### Immunofluorescence staining

LUAD cells (10 × 10^3^) expressing *TK1*, *DCK*, or NS shRNA were plated onto coverslips in a multi-well chambered slide. After 24 h, cells were washed with PBS and fixed with 3.7% paraformaldehyde with 2% sucrose. Cells were then permeabilized using 0.3% Triton X-100. After washing again with PBS, slides were blocked using 5% BSA in PBS and then incubated with primary antibodies (phospho-γ-H2AX or vinculin) diluted in 5% BSA in PBS (1:200) (see S4 Table) for 2 h at room temperature. After another wash, cells were incubated with secondary antibodies (AlexaFluor-488 anti-rabbit or anti-mouse, 1:1,000) diluted in 5% BSA in PBS (see S4 Table) for 1 h at room temperature. Lastly, the cells were stained with DAPI and mounted onto glass slides. Fluorescence images were acquired using a LEICA SP5 Confocal Laser Scanning Microscope. The same procedure was performed with A549 cells expressing either *TK1* shRNA or control, NS shRNA to validate the specificity of the TK1 antibody used for immunohistochemistry. The specificity of the antibody was also validated by a separate immunoblot analysis.

### Measurement of Rho GTPase activity

GST-Rhotekin-RBD was purified as described previously[70]. For the measurement of Rho GTPase activity, approximately 3 × 10^6^ cells were plated in 100-mm cell culture dishes and allowed to grow for 48 h. The cells were collected by scraping and then lysed in IP lysis buffer. Protein concentrations were determined using Bradford Protein Assay Reagent (Bio-Rad Laboratories, Hercules, CA, USA). Equal amounts of protein (500 μg) were then aliquoted, and each sample was incubated with 50 μl GST-Rhotekin-RBD agarose beads at 4°C for 4 h. Complexes containing RBD-bound RhoA were centrifuged at 3,000 rpm for 5 min, and the pellets were washed twice with IP lysis buffer. RBD-bound RhoA proteins were separated by boiling the sample in 2× protein-loading buffer for 5 min, and supernatants containing 50 μg protein were used as the input for the pull-down assays described above. The resulting samples were electrophoresed on 12% SDS-PAGE gels, and both active and total RhoA levels were detected by immunoblot.

### Measurement of cellular GTP/GDP using HPLC‐MS/MS analysis

LUAD cells expressing *TK1* or NS shRNA were analyzed for GTP/GDP alterations in metabolic pathways using high-performance liquid chromatography-tandem mass spectrometry (HPLC‐MS/MS). Briefly, 10^6^ cells for each condition were analyzed in duplicate. Samples were prepared by mixing cells with 4 ml methanol, 2 ml chloroform, and 2 ml water in an 8-ml glass vial. This formed a two-layered system. The top layer, containing water and methanol, was removed, dried, and resuspended in 200μL 80% acetonitrile. The samples were then analyzed on a Thermo Ultimate 3000 LC, coupled with a Q‐Exactive Plus mass spectrometer, with 5 μL of each sample injected onto a Zic‐pHILIC Column (150 × 2.1 mm, 5-micron particles, EMD Millipore). The mobile phases were (A) 20 mM ammonium carbonate in 0.1% ammonium hydroxide and (B) acetonitrile 97% in water. The gradient conditions were as follow: 100% B at 0 min, 40% B at 20 min, 0% B at 30 min, for 5 min, then back to 100% B in 5 min, followed by 10 min of re‐equilibration. Data were obtained in both positive and negative ion modes. The positive ion mode data were more intense for GTP and GDP, and therefore these were used for relative quantification. Structural confirmation was performed through high-resolution accurate mass measurement, high-resolution MS/MS measurement, and retention time comparison with standard.

### Mouse tumorigenesis experiments with cells expressing *TK1* shRNA

Athymic nude (NU/J) mice (Stock No. 002019, Jackson Laboratory), aged 4–5 weeks, were injected subcutaneously with 5 × 10^6^ LUAD cells expressing *TK1* shRNA or NS shRNA. Tumor volume was measured every 3 days and was calculated using the following formula: length × width^2^ × 0.5.

### Tail vein injection of cells expressing *TK1* shRNAs

A549 cells stably expressing firefly luciferase under control of the cytomegalovirus (CMV) promoter were generated by co-transfecting the transposon vector, piggyBac GFP-Luc, and the helper plasmid, Act-PBase, as described previously[71]. Cells with stable transposon integration were selected using blasticidin S (Invitrogen, Thermo Fisher Scientific). A549-GFP-*F-Luc* cells (2.5 × 10^5^) expressing *TK1* shRNAs or NS shRNA were then injected into NSG mice (Stock No. 005557, Jackson Laboratory) *via* the tail vein. Mice were imaged using the IVIS Spectrum In Vivo Imaging System (Perkin Elmer), and total luminescence counts of tumor-bearing areas were measured using Living Image *in vivo* imaging software (Perkin Elmer).

### Statistical analysis

All experiments were conducted with at least three biological replicates. For the measurement of cellular GTP/GDP by HPLC‐MS/MS, two biological replicates were employed. Results for individual experiments were expressed as the mean ± standard error of the mean (SEM). For the analysis of tumor progression in mice, statistical assessment was performed using the area under the curve (AUC) method on GraphPad Prism, version 8.0 for Macintosh (GraphPad Software, San Diego, California, USA; www.graphpad.com). To analyze the correlation of the mRNA expression levels of *TK1* and *MAZ*, we downloaded the expression data for TK1 and MAZ from the Okayama lung, Stearman lung, and Su lung datasets [22, 25, 26]. We calculated the Pearson correlation coefficients for each dataset using GraphPad Prism, version 8.0 for Macintosh (GraphPad Software, San Diego, California, USA; www.graphpad.com). The *P*-values for all other experiments were calculated using the two-tailed unpaired Student’s *t*-test on GraphPad Prism, version 8.0 for Macintosh (GraphPad Software, San Diego, California, USA; www.graphpad.com). ns, *, **, ***, and **** indicate non-significant *P*-value, *P* < 0.05, < 0.01, < 0.001, and < 0.0001, respectively.

## Supporting information

S1 Fig *TK1* is upregulated in lung adenocarcinoma. **(A)** Lung adenocarcinoma (LUAD) datasets were analyzed for *TK1* mRNA expression. Average *TK1* expression in patient-derived LUAD samples relative to normal lung tissues is shown. **(B)** Plot showing average relative *TK1* mRNA expression for living *vs*. deceased patients in LUAD datasets. *P*-value for the comparison is shown. **(C)** Validation of the specificity of the TK1 antibody used for immunohistochemistry by immunoblot by analyzing A549 cells expressing either non- *TK1* shRNAs or non-specific (NS) shRNA. **(D)** Validation of the specificity of the TK1 antibody used for immunohistochemistry by immunofluorescence in A549 cells expressing either *TK1* shRNAs or non-specific (NS) shRNA using DAPI (blue)/TK1 (green) immunofluorescence and confocal microscopy. Scale bar, 20 μm for top images, and 10 μm for magnified images at the bottom.
(TIF)

S2 Fig Validation of *TK1* knockdown, clonogenic assay, and MTT assay in LUAD cell lines. (A) *TK1* mRNA expression was measured by quantitative reverse transcriptase-PCR (qRT-PCR) in LUAD cell lines expressing either short hairpin RNAs (shRNAs) targeting *TK1* or non-specific (NS) shRNA control. *TK1* expression in *TK1* shRNA-expressing cells is plotted relative to that in NS shRNA-expressing cells. (B) Clonogenic assay of LUAD cells expressing either *TK1* shRNA or NS shRNA. Representative images are shown. (C) MTT assays of LUAD cells expressing either *TK1* shRNA or NS shRNA 20 h after plating. Relative cell proliferation is shown. Data are presented as the mean ± standard error of the mean (SEM); ns = not significant. *** represents *P* < 0.001.
(TIF)

S3 Fig MAZ is transcriptionally regulated by the MAPK pathway in LAUD cells. LUAD cell lines were treated with trametinib (250 nM) or dimethyl sulfoxide (DMSO) control for 24 h, and mRNA levels of the indicated transcription factors were measured by qRT-PCR. Expression in cells treated with trametinib is plotted relative to that in DMSO-treated cells. Data are presented as the mean ± SEM; ns = not significant. *, **, ***, and **** represent *P* < 0.05, *P* < 0.01, *P* < 0.001, and *P* < 0.0001, respectively.
(TIF)

S4 Fig Analysis of Pearson correlation coefficients in LUAD sample datasets. **(A-C)** Pearson correlation coefficient was calculated for *TK1* and *MAZ* mRNA expression levels in the indicated datasets. Results are presented using GraphPad Prism, version 8.0. Pearson coefficient (r), 95% confidence interval, R-squared, and *P*-values are shown.
(TIF)

S5 Fig *TK1* knockdown-induced DNA damage is not required for inhibition of LUAD tumor growth. **(A)** (Left) DNA damage was measured in the indicated LUAD cell lines expressing *TK1* shRNA or control, NS shRNA using phospho-γ-H2AX immunofluorescence and confocal microscopy. Representative images are shown. Scale bar, 20 μm. (Right) Relative intensity of phospho-γ-H2AX staining in the indicated LUAD cell lines expressing *TK1* shRNA or NS shRNA in the left panel. **(B)** *DCK* mRNA expression was measured by qRT-PCR in A549 cells expressing either *DCK* shRNA or control, NS shRNA. *DCK* expression in *DCK* shRNA-expressing cells is plotted relative to that in NS shRNA-expressing cells. **(C)** DCK protein levels were measured by immunoblotting in A549 cells expressing *DCK* shRNA or NS shRNA. ACTINB was used as a loading control. **(D)** (Left) DNA damage was measured in A549 cells expressing *DCK* shRNA or NS shRNA using phospho-γ-H2AX immunofluorescence and confocal microscopy. Representative images are shown. Scale bar, 20 μm. (Right) Relative intensity of phospho-γ-H2AX staining in A549 cells expressing *DCK* shRNA or NS shRNA in the left panel. **(E)** (Left) Anchorage-independent growth was measured by soft-agar assay in A549 cells expressing either *DCK* shRNA or NS shRNA. Representative images of soft-agar colonies of A549 cells expressing either *DCK* shRNA or NS shRNA are shown. Scale bar, 500 μm. (Right) Plot showing relative colony sizes in the soft-agar assay on the left. **(F)** (Left) Wound-healing assays of A549 cells expressing *DCK* shRNA or NS shRNA. Representative images at the indicated times are shown. Scale bar, 200 μm. (Right) Relative migration (%) calculated from the data presented on the left. **(G)** (Top) Matrigel invasion assays with the indicated A549 cell lines expressing *DCK* shRNA or NS shRNA; representative images are shown. Scale bar, 200 μm. (Bottom) Relative invasion (%) in Matrigel assays shown in the top panel. Data are presented as the mean ± SEM. ns = not significant. *, **, and *** represent *P* < 0.05, *P* < 0.01, and *P* < 0.001, respectively.
(TIF)

S6 Fig Expression of *DCK* mRNA in lung adenocarcinoma. **(A-D)** The indicated lung adenocarcinoma datasets were analyzed for *DCK* mRNA expression. Relative *DCK* expression in patient-derived LUAD samples compared to normal lung tissues is shown. No significant up- or downregulation of *DCK* in LUAD compared to normal tissue was observed.
(TIF)

S7 Fig Role of DTYMK and NME1 in lung adenocarcinoma. **(A)** Schematic showing the enzymatic steps leading to the generation of dTTP and dGDP. **(B)** A549 cells expressing *DTYMK* shRNA or *NME1* shRNA, or the respective NS shRNA controls, were analyzed by qRT-PCR for the expression of *DTYMK* and *NME1* mRNA, respectively. Expression in *DTYMK* or *NME1* shRNA-expressing cells is plotted relative to that in NS shRNA-expressing cells. **(C)** (Left) Anchorage-independent growth was measured by soft-agar assay in A549 cells expressing either *DTYMK* or *NME1* shRNAs, or the respective NS shRNA controls. Representative images of soft-agar colonies from indicated conditions are shown. (Right) Plot showing relative colony sizes (%) from the soft-agar assay shown on the left. **(D)** Active RhoA was measured by GST pull-down assay and immunoblot analysis in A549 cells expressing *DTYMK* shRNA or NS shRNA control. GST-RBD was used as a control in the pull-down assay, and total RhoA in whole-cell lysates was used as a loading control for immunoblot analysis. **(E)** Active RhoA was measured by GST pull-down assay and immunoblot analysis in A549 cells expressing *NME1* shRNA or NS shRNA control. GST-RBD was used as a control in the pull-down assay, and total RhoA in whole-cell lysates was used as a loading control for immunoblot analysis. Data are presented as the mean ± SEM; **, ***, and **** represent *P* < 0.01, *P* < 0.001, and *P* < 0.0001, respectively.
(TIF)

S8 Fig Validation of microarray data, effect of candidate-gene knockdown on anchorage-independent growth, and effect of *GDF15* knockdown on RhoA GTPase activity. **(A)** Expression of the indicated genes was measured by qRT-PCR in A549 cells expressing either *TK1* shRNA or NS shRNA control. Expression in *TK1* shRNA-expressing cells is plotted relative to that in NS shRNA-expressing cells. **(B)** Expression of *HMGB3*, *MMD*, *HIPK2*, and *HILPDA* was measured in A549 cells expressing shRNAs to *HMGB3*, *MMD*, *HIPK2*, and *HILPDA*, respectively, or the NS shRNA control. Expression in *HMGB3*, *MMD*, *HIPK2*, and *HILPDA* shRNA-expressing cells is plotted relative to that NS shRNA-expressing cells. **(C)** (Top) Anchorage-independent growth was measured by soft-agar assay in A549 cells expressing shRNAs to *HMGB3*, *MMD*, *HIPK2*, or *HILPDA*, or a NS shRNA control. Representative images of soft-agar colonies from knockdown and control cells are shown. Scale bar, 500 μm. (Bottom) Relative colony sizes from the soft-agar assay shown in top panel. **(D)** Active RhoA was measured by GST pull-down assay and immunoblot analysis in A549 cells expressing *GDF15* shRNA or NS shRNA. GST-RBD was used as a control in the pull-down assay. Total RhoA in whole-cell lysates was used as a loading control for immunoblot analysis. Data are presented as the mean ± SEM; ns = not significant. *, **, ***, and **** represent *P* < 0.05, *P* < 0.01, *P* < 0.001, and *P* < 0.0001, respectively.
(TIF)

S1 Table Summary of immunohistochemistry staining for TK1 in human patient-derived LUAD samples and matched normal adjacent lung tissues. (DOCX)

S2 Table Analysis of transcription factors on *TK1* promoter by PROMO 3.0 and rVISTA 2.0. (DOCX)

S3 Table Fold change for significantly altered genes in A549 cells expressing *TK1* shRNAs compared to the cells expressing non-silencing shRNA. (DOCX)

S4 Table Primer sequences for RT-qPCR analysis; clone ID and catalog numbers for shRNAs (Open Biosystems); antibodies used; source and concentration of chemical inhibitors used. (DOCX)
