# Supplementary figures and images for: Loss of thymidine kinase 1 inhibits lung cancer growth and metastatic attributes by reducing GDF15 expression
Source: PLoS Genet. 2019 Oct 7;15(10):e1008439. doi: 10.1371/journal.pgen.1008439 (PMC6797230; doi:10.1371/journal.pgen.1008439)

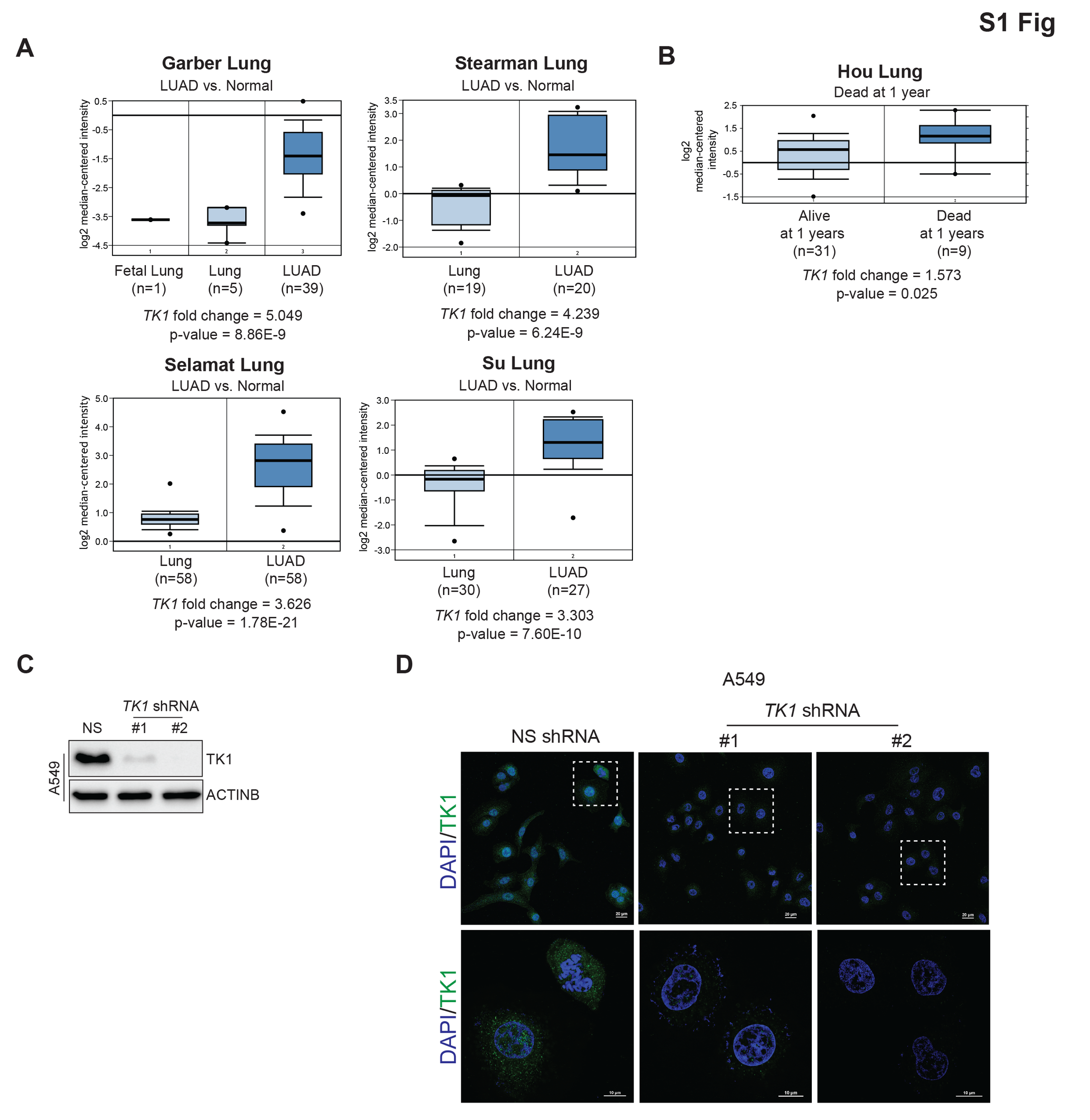

Supplement: S1 Fig — (A) Lung adenocarcinoma (LUAD) datasets were analyzed for TK1 mRNA expression. Average TK1 expression in patient-derived LUAD samples relative to normal lung tissues is shown. (B) Plot showing average relative TK1 mRNA expression for living vs. deceased patients in LUAD datasets. P-value for the comparison is shown. (C) Validation of the specificity of the TK1 antibody used for immunohistochemistry by immunoblot by analyzing A549 cells expressing either non- TK1 shRNAs or non-specific (NS) shRNA. (D) Validation of the specificity of the TK1 antibody used for immunohistochemistry by immunofluorescence in A549 cells expressing either TK1 shRNAs or non-specific (NS) shRNA using DAPI (blue)/TK1 (green) immunofluorescence and confocal microscopy. Scale bar, 20 μm for top images, and 10 μm for magnified images at the bottom. (TIF) [file pgen.1008439.s001.tif]

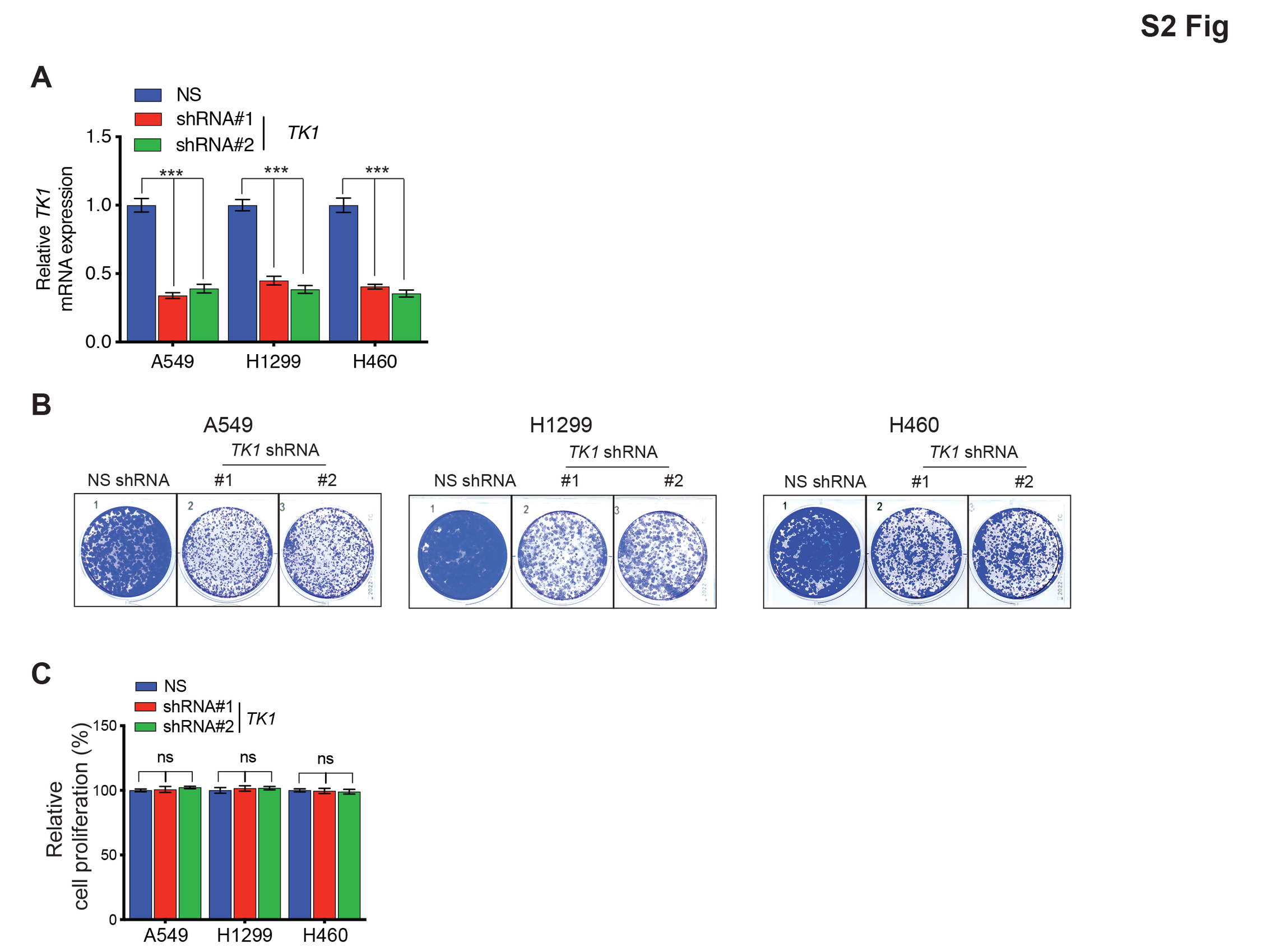

Supplement: S2 Fig — (A) TK1 mRNA expression was measured by quantitative reverse transcriptase-PCR (qRT-PCR) in LUAD cell lines expressing either short hairpin RNAs (shRNAs) targeting TK1 or non-specific (NS) shRNA control. TK1 expression in TK1 shRNA-expressing cells is plotted relative to that in NS shRNA-expressing cells. (B) Clonogenic assay of LUAD cells expressing either TK1 shRNA or NS shRNA. Representative images are shown. (C) MTT assays of LUAD cells expressing either TK1 shRNA or NS shRNA 20 h after plating. Relative cell proliferation is shown. Data are presented as the mean ± standard error of the mean (SEM); ns = not significant. *** represents P < 0.001. (TIF) [file pgen.1008439.s002.tif]

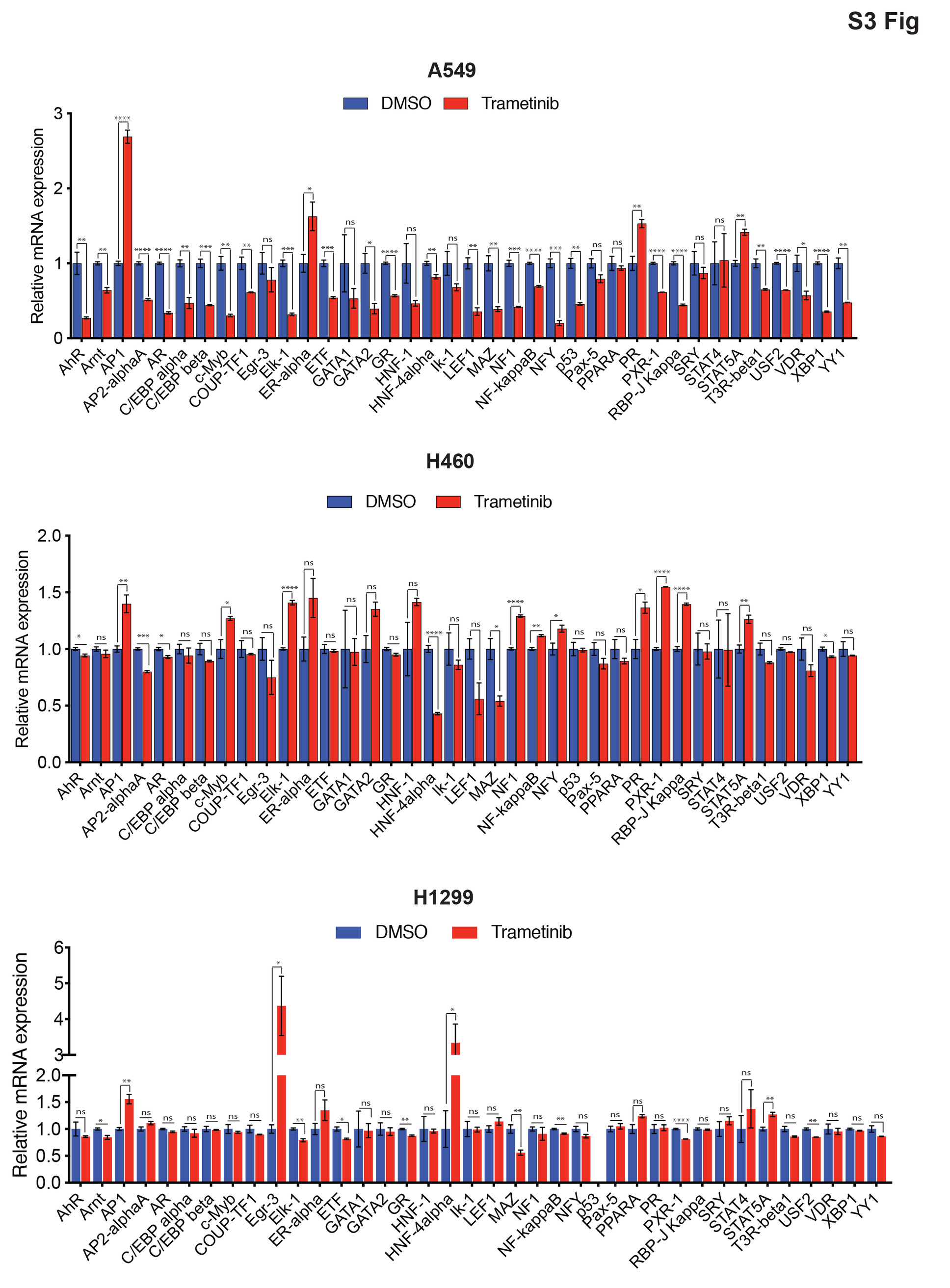

Supplement: S3 Fig — LUAD cell lines were treated with trametinib (250 nM) or dimethyl sulfoxide (DMSO) control for 24 h, and mRNA levels of the indicated transcription factors were measured by qRT-PCR. Expression in cells treated with trametinib is plotted relative to that in DMSO-treated cells. Data are presented as the mean ± SEM; ns = not significant. *, **, ***, and **** represent P < 0.05, P < 0.01, P < 0.001, and P < 0.0001, respectively. (TIF) [file pgen.1008439.s003.tif]

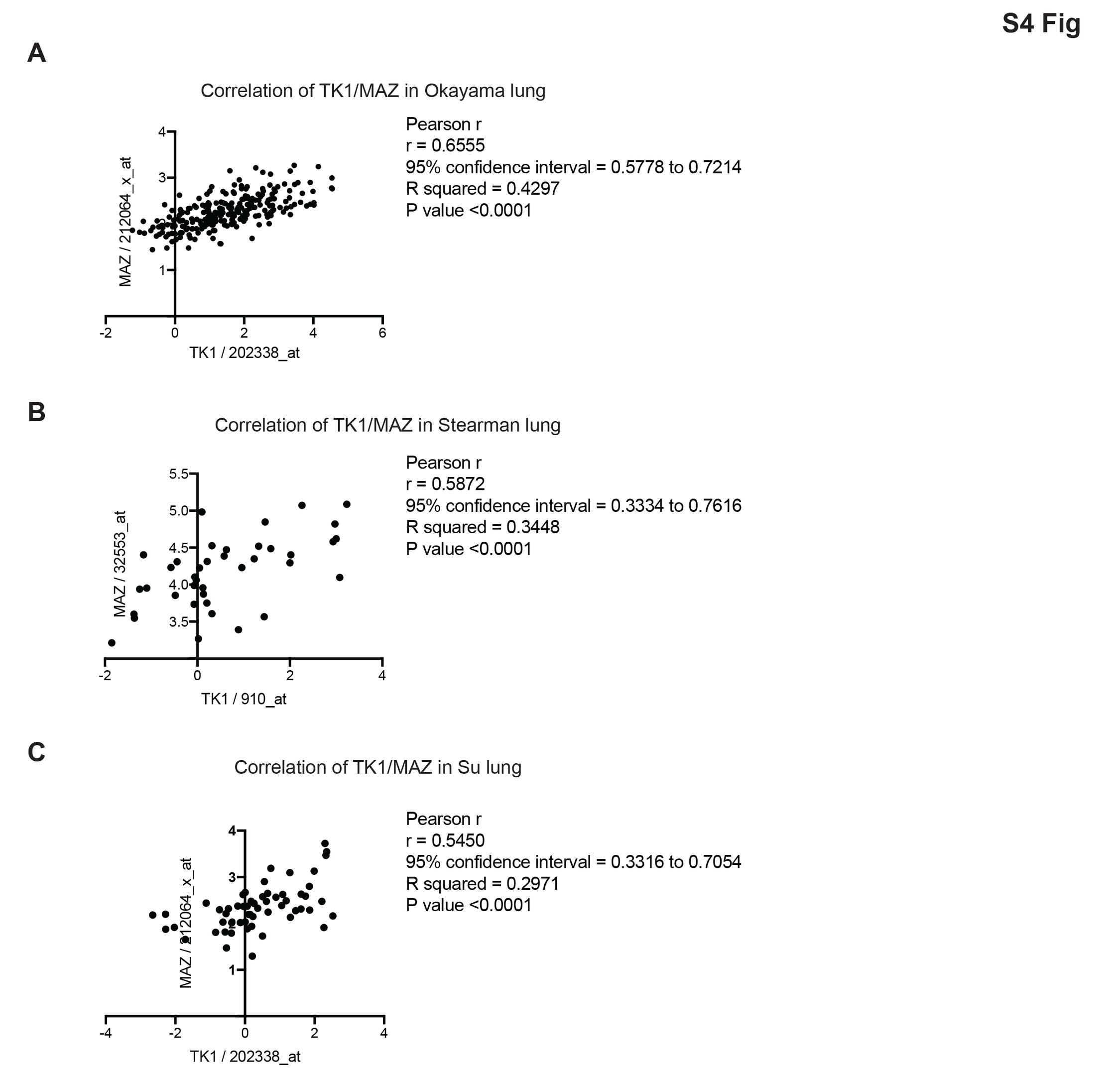

Supplement: S4 Fig — (A-C) Pearson correlation coefficient was calculated for TK1 and MAZ mRNA expression levels in the indicated datasets. Results are presented using GraphPad Prism, version 8.0. Pearson coefficient (r), 95% confidence interval, R-squared, and P-values are shown. (TIF) [file pgen.1008439.s004.tif]

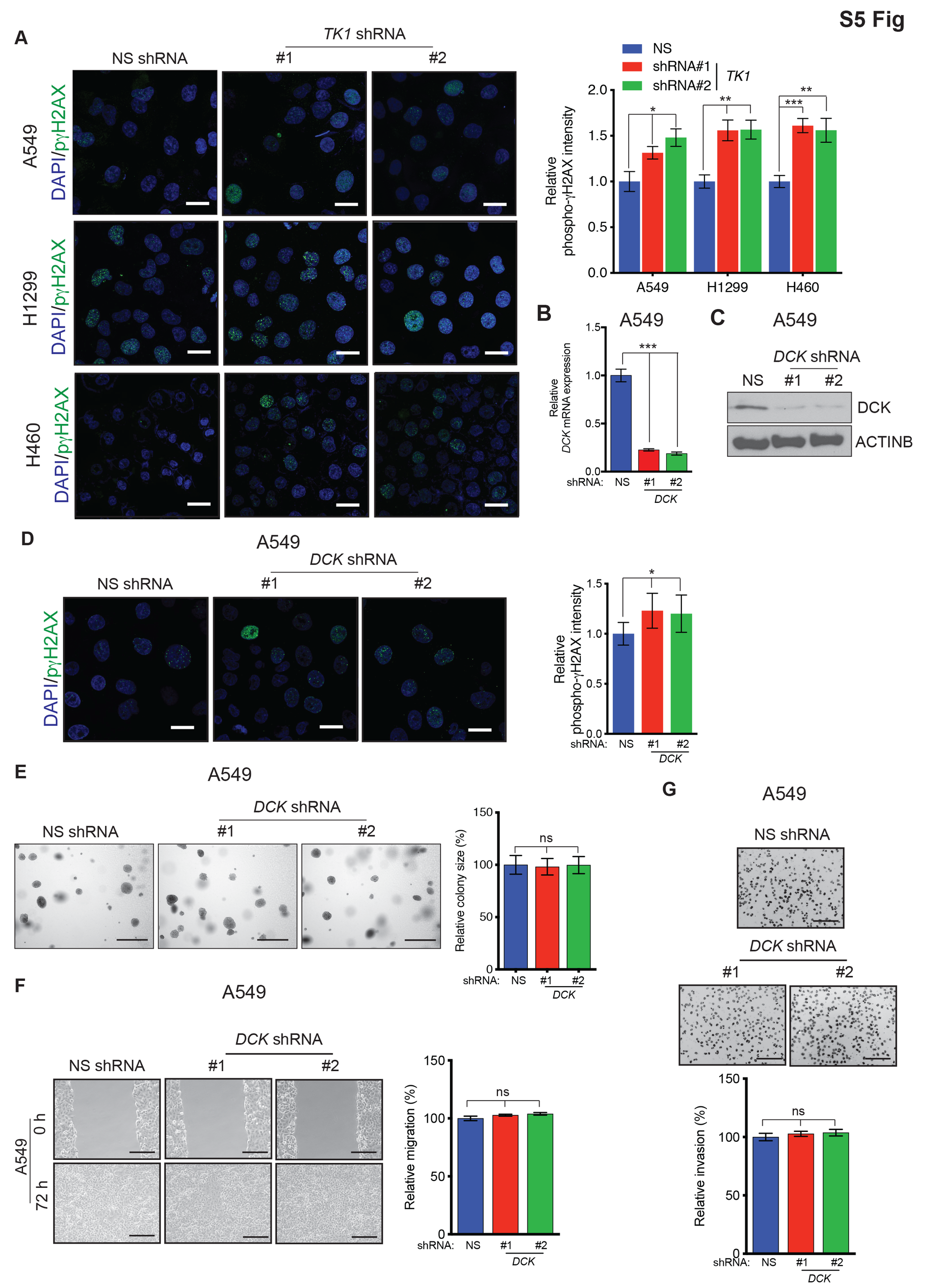

Supplement: S5 Fig — (A) (Left) DNA damage was measured in the indicated LUAD cell lines expressing TK1 shRNA or control, NS shRNA using phospho-γ-H2AX immunofluorescence and confocal microscopy. Representative images are shown. Scale bar, 20 μm. (Right) Relative intensity of phospho-γ-H2AX staining in the indicated LUAD cell lines expressing TK1 shRNA or NS shRNA in the left panel. (B) DCK mRNA expression was measured by qRT-PCR in A549 cells expressing either DCK shRNA or control, NS shRNA. DCK expression in DCK shRNA-expressing cells is plotted relative to that in NS shRNA-expressing cells. (C) DCK protein levels were measured by immunoblotting in A549 cells expressing DCK shRNA or NS shRNA. ACTINB was used as a loading control. (D) (Left) DNA damage was measured in A549 cells expressing DCK shRNA or NS shRNA using phospho-γ-H2AX immunofluorescence and confocal microscopy. Representative images are shown. Scale bar, 20 μm. (Right) Relative intensity of phospho-γ-H2AX staining in A549 cells expressing DCK shRNA or NS shRNA in the left panel. (E) (Left) Anchorage-independent growth was measured by soft-agar assay in A549 cells expressing either DCK shRNA or NS shRNA. Representative images of soft-agar colonies of A549 cells expressing either DCK shRNA or NS shRNA are shown. Scale bar, 500 μm. (Right) Plot showing relative colony sizes in the soft-agar assay on the left. (F) (Left) Wound-healing assays of A549 cells expressing DCK shRNA or NS shRNA. Representative images at the indicated times are shown. Scale bar, 200 μm. (Right) Relative migration (%) calculated from the data presented on the left. (G) (Top) Matrigel invasion assays with the indicated A549 cell lines expressing DCK shRNA or NS shRNA; representative images are shown. Scale bar, 200 μm. (Bottom) Relative invasion (%) in Matrigel assays shown in the top panel. Data are presented as the mean ± SEM. ns = not significant. *, **, and *** represent P < 0.05, P < 0.01, and P < 0.001, respectively. (TIF) [file pgen.1008439.s005.tif]

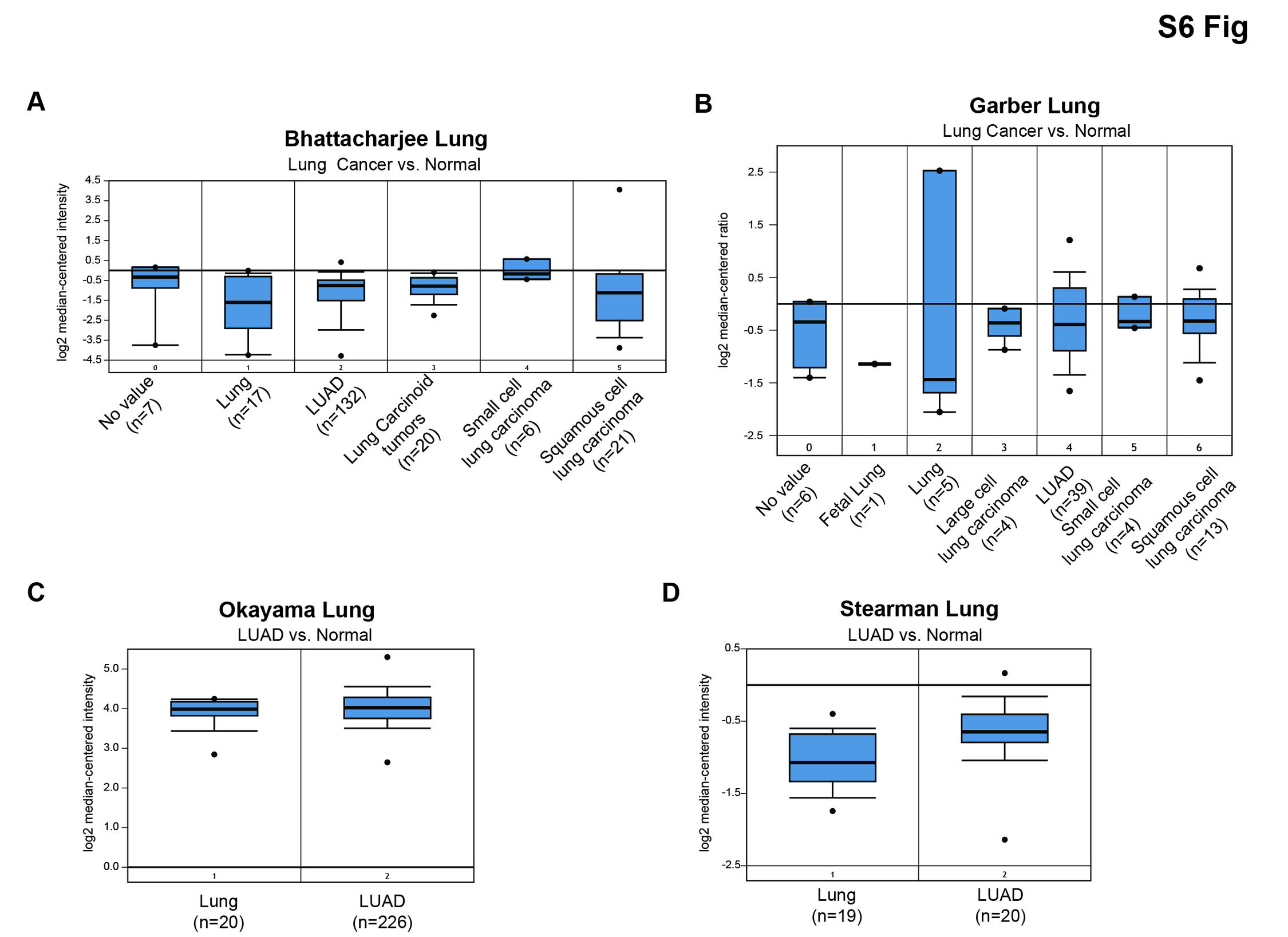

Supplement: S6 Fig — (A-D) The indicated lung adenocarcinoma datasets were analyzed for DCK mRNA expression. Relative DCK expression in patient-derived LUAD samples compared to normal lung tissues is shown. No significant up- or downregulation of DCK in LUAD compared to normal tissue was observed. (TIF) [file pgen.1008439.s006.tif]

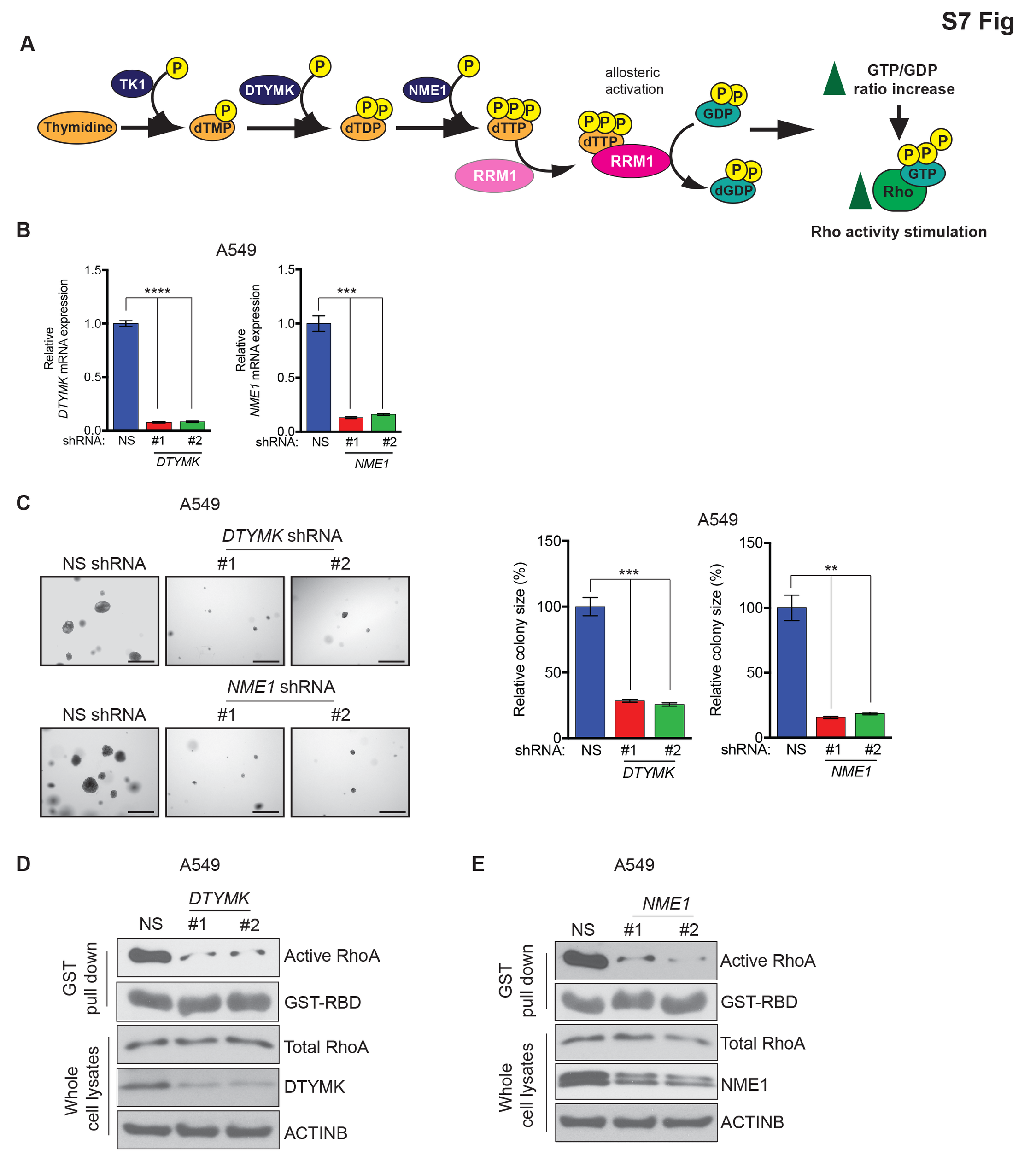

Supplement: S7 Fig — (A) Schematic showing the enzymatic steps leading to the generation of dTTP and dGDP. (B) A549 cells expressing DTYMK shRNA or NME1 shRNA, or the respective NS shRNA controls, were analyzed by qRT-PCR for the expression of DTYMK and NME1 mRNA, respectively. Expression in DTYMK or NME1 shRNA-expressing cells is plotted relative to that in NS shRNA-expressing cells. (C) (Left) Anchorage-independent growth was measured by soft-agar assay in A549 cells expressing either DTYMK or NME1 shRNAs, or the respective NS shRNA controls. Representative images of soft-agar colonies from indicated conditions are shown. (Right) Plot showing relative colony sizes (%) from the soft-agar assay shown on the left. (D) Active RhoA was measured by GST pull-down assay and immunoblot analysis in A549 cells expressing DTYMK shRNA or NS shRNA control. GST-RBD was used as a control in the pull-down assay, and total RhoA in whole-cell lysates was used as a loading control for immunoblot analysis. (E) Active RhoA was measured by GST pull-down assay and immunoblot analysis in A549 cells expressing NME1 shRNA or NS shRNA control. GST-RBD was used as a control in the pull-down assay, and total RhoA in whole-cell lysates was used as a loading control for immunoblot analysis. Data are presented as the mean ± SEM; **, ***, and **** represent P < 0.01, P < 0.001, and P < 0.0001, respectively. (TIF) [file pgen.1008439.s007.tif]

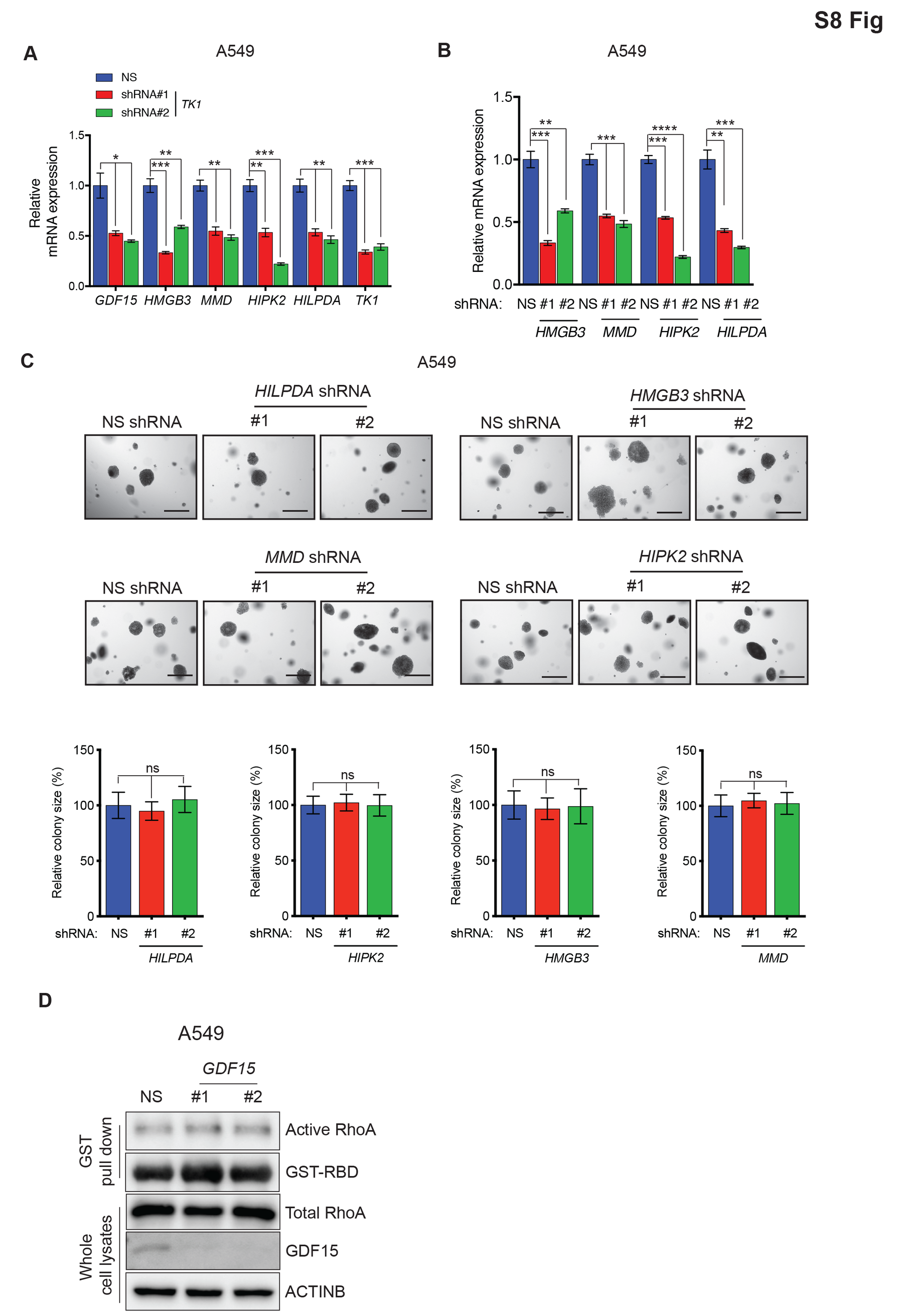

Supplement: S8 Fig — (A) Expression of the indicated genes was measured by qRT-PCR in A549 cells expressing either TK1 shRNA or NS shRNA control. Expression in TK1 shRNA-expressing cells is plotted relative to that in NS shRNA-expressing cells. (B) Expression of HMGB3, MMD, HIPK2, and HILPDA was measured in A549 cells expressing shRNAs to HMGB3, MMD, HIPK2, and HILPDA, respectively, or the NS shRNA control. Expression in HMGB3, MMD, HIPK2, and HILPDA shRNA-expressing cells is plotted relative to that NS shRNA-expressing cells. (C) (Top) Anchorage-independent growth was measured by soft-agar assay in A549 cells expressing shRNAs to HMGB3, MMD, HIPK2, or HILPDA, or a NS shRNA control. Representative images of soft-agar colonies from knockdown and control cells are shown. Scale bar, 500 μm. (Bottom) Relative colony sizes from the soft-agar assay shown in top panel. (D) Active RhoA was measured by GST pull-down assay and immunoblot analysis in A549 cells expressing GDF15 shRNA or NS shRNA. GST-RBD was used as a control in the pull-down assay. Total RhoA in whole-cell lysates was used as a loading control for immunoblot analysis. Data are presented as the mean ± SEM; ns = not significant. *, **, ***, and **** represent P < 0.05, P < 0.01, P < 0.001, and P < 0.0001, respectively. (TIF) [file pgen.1008439.s008.tif]
