# Supplementary material for: Loss of thymidine kinase 1 inhibits lung cancer growth and metastatic attributes by reducing GDF15 expression
Source: PLoS Genet. 2019 Oct 7;15(10):e1008439. doi: 10.1371/journal.pgen.1008439 (PMC6797230; doi:10.1371/journal.pgen.1008439)
Supplement: S1 Table — (DOCX) [file pgen.1008439.s009.docx]

**S1 Table**. Summary of immunohistochemistry staining for TK1 in human patient-derived LUAD samples and matched normal adjacent lung tissues.

| **Staining** | | **Cytoplasmic** | | | | | | | | | | | | | | | | | | | |
| --- | --- | --- | --- | --- | --- | --- | --- | --- | --- | --- | --- | --- | --- | --- | --- | --- | --- | --- | --- | --- | --- |
| Density | | 0 | | | | | 1+ | | | | | 2+ | | | | | 3+ | | | | |
| Percentile | | 0 = Less than 10% | | | | | 1+ = 10-25% | | | | | 2+ = 26-50% | | | | | 3+ = More than 50% | | | | |
|  | | | | | | | | | | | | | | | | | | | | | |
|  | | | | | | | | | | | | | | | | | | | | | |
| **Tissue** | **LUAD** | | | | | | | | | | | | | **Matched normal adjacent lung tissue** | | | | | | | |
| **No.** | **Sex** | | **Age** | **Organ** | **Pathology diagnosis** | **Grade** | | **Stage** | **pTNM** | **Type** | **Density** | | **Percentile** | **Pathology diagnosis** | **Grade** | **Stage** | | **pTNM** | **Type** | **Density** | **Percentile** |
| 1 | F | | 42 | Lung | Adenocarcinoma | 2 | | IIIB | T4N1M0 | Malignant | 2+ | | 3+ | Adjacent normal lung tissue | – | – | | – | NAT | 2+ | 1+ |
| 2 | F | | 39 | Lung | Adenocarcinoma | 2 | | I | T2N0M0 | Malignant | 2+ | | 3+ | Adjacent normal lung tissue | – | – | | – | NAT | 1+ | 1+ |
| 3 | M | | 50 | Lung | Adenocarcinoma | 3 | | IIB | T3N0M0 | Malignant | 2+ | | 3+ | Emphysema | – | – | | – | NAT | 1+ | 3+ |
| 4 | M | | 75 | Lung | Adenocarcinoma with necrosis | 2 | | I | T2N0M0 | Malignant | 3+ | | 3+ | Adjacent normal lung tissue | – | – | | – | NAT | 1+ | 2+ |
| 5 | F | | 59 | Lung | Adenocarcinoma | 2 | | IIB | T2N1M0 | Malignant | 3+ | | 3+ | Adjacent normal lung tissue | – | – | | – | NAT | 1+ | 2+ |
| 6 | M | | 62 | Lung | Adenocarcinoma | 2 | | IV | T2N0M1 | Malignant | 2+ | | 3+ | Adjacent normal lung tissue | – | – | | – | NAT | 1+ | 2+ |
| 7 | M | | 51 | Lung | Adenocarcinoma | 2 | | IIA | T2N1M0 | Malignant | 2+ | | 3+ | Adjacent normal lung tissue | – | – | | – | NAT | 1+ | 2+ |
| 8 | M | | 49 | Lung | Adenocarcinoma | 3 | | II | T1N1M0 | Malignant | 2+ | | 3+ | Adjacent normal lung tissue | – | – | | – | NAT | 1+ | 1+ |
| 9 | M | | 59 | Lung | Adenocarcinoma | 2 | | II | Y2N1M0 | Malignant | 3+ | | 3+ | Adjacent normal lung tissue | – | – | | – | NAT | 1+ | 1+ |
| 10 | M | | 65 | Lung | Adenocarcinoma | 2 | | IIIA | T3N2M0 | Malignant | 2+ | | 2+ | Pulmonary collapse | – | – | | – | NAT | 1+ | 2+ |
| 11 | F | | 54 | Lung | Adenocarcinoma | 3 | | IIIA | T3N1M0 | Malignant | 2+ | | 3+ | Pulmonary collapse | – | – | | – | NAT | 1+ | 2+ |
| 12 | F | | 59 | Lung | Adenocarcinoma with necrosis | 2 | | I | T2N0M0 | Malignant | 2+ | | 3+ | Adjacent normal lung tissue | – | – | | – | NAT | 1+ | 2+ |
| 13 | F | | 65 | Lung | Adenocarcinoma | 2 | | I | T2N0M0 | Malignant | 2+ | | 3+ | Adjacent normal lung tissue | – | – | | – | NAT | 1+ | 2+ |
| 14 | M | | 51 | Lung | Adenocarcinoma | 2 | | I | T1N0M0 | Malignant | 3+ | | 3+ | Adjacent normal lung tissue | – | – | | – | NAT | 1+ | 2+ |
| 15 | F | | 37 | Lung | Adenocarcinoma | 2 | | I | T2N0M0 | Malignant | 2+ | | 3+ | Adjacent normal lung tissue | – | – | | – | NAT | 1+ | 3+ |
| 16 | F | | 52 | Lung | Adenocarcinoma | 2 | | II | T2N1M0 | Malignant | 3+ | | 3+ | Adjacent normal lung tissue | – | – | | – | NAT | 2+ | 2+ |
| 17 | F | | 52 | Lung | Adenocarcinoma | 2 | | II | T2N1M0 | Malignant | 3+ | | 3+ | Adjacent normal lung tissue | – | – | | – | NAT | 1+ | 2+ |
| 18 | M | | 62 | Lung | Adenocarcinoma | 2 | | I | T2N0M0 | Malignant | 2+ | | 3+ | Adjacent normal lung tissue | – | – | | – | NAT | 1+ | 1+ |
| 19 | F | | 49 | Lung | Adenocarcinoma | 2 | | I | T2N0M0 | Malignant | 2+ | | 3+ | Adjacent normal lung tissue | – | – | | – | NAT | 1+ | 1+ |
| 20 | F | | 42 | Lung | Adenocarcinoma | 2 | | I | T2N0M0 | Malignant | 2+ | | 3+ | Adjacent normal lung tissue | – | – | | – | NAT | 1+ | 2+ |
| 21 | M | | 55 | Lung | Adenocarcinoma | 2 | | I | T2N0M0 | Malignant | 1+ | | 2+ | Emphysema | – | – | | – | NAT | 1+ | 1+ |
| 22 | M | | 70 | Lung | Adenocarcinoma with necrosis | 2 | | I | T2N0M0 | Malignant | 2+ | | 3+ | Adjacent normal lung tissue | – | – | | – | NAT | 1+ | 2+ |
| 23 | M | | 52 | Lung | Adenocarcinoma | 3 | | II | T2N1M0 | Malignant | 3+ | | 3+ | Adjacent normal lung tissue | – | – | | – | NAT | 1+ | 2+ |
| 24 | F | | 37 | Lung | Adenocarcinoma | 3 | | I | T2N0M0 | Malignant | 3+ | | 3+ | Adjacent normal lung tissue | – | – | | – | NAT | 2+ | 2+ |
| 25 | F | | 63 | Lung | Adenocarcinoma | 2 | | I | T2N0M0 | Malignant | 3+ | | 3+ | Adjacent normal lung tissue | – | – | | – | NAT | 2+ | 2+ |
| 26 | F | | 64 | Lung | Adenocarcinoma | 3 | | I | T2N0M0 | Malignant | 3+ | | 3+ | Adjacent normal lung tissue with tumor embolus | – | – | | – | NAT | 2+ | 2+ |
| 27 | M | | 70 | Lung | Adenocarcinoma | 3 | | I | T2N0M0 | Malignant | 2+ | | 3+ | Adjacent normal lung tissue | – | – | | – | NAT | 2+ | 2+ |
| 28 | M | | 40 | Lung | Adenocarcinoma | 3 | | I | T2N0M0 | Malignant | 1+ | | 3+ | Adjacent normal lung tissue | – | – | | – | NAT | 1+ | 1+ |
| 29 | F | | 62 | Lung | Adenocarcinoma | 3 | | I | T2N0M0 | Malignant | 2+ | | 3+ | Adjacent normal lung tissue | – | – | | – | NAT | 1+ | 1+ |
| 30 | M | | 64 | Lung | Adenocarcinoma | 3 | | I | T2N0M0 | Malignant | 2+ | | 3+ | Adjacent normal lung tissue | – | – | | – | NAT | 1+ | 2+ |
| 31 | M | | 63 | Lung | Adenocarcinoma | 3 | | I | T2N0M0 | Malignant | 1+ | | 3+ | Adjacent normal lung tissue | – | – | | – | NAT | 1+ | 1+ |
| 32 | M | | 58 | Lung | Adenocarcinoma | 2 | | I | T2N0M0 | Malignant | 2+ | | 3+ | Adjacent normal lung tissue | – | – | | – | NAT | 1+ | 3+ |
| 33 | F | | 58 | Lung | Adenocarcinoma | 3 | | I | T2N0M0 | Malignant | 2+ | | 3+ | Adjacent normal lung tissue | – | – | | – | NAT | 1+ | 2+ |
| 34 | F | | 32 | Lung | Adenocarcinoma | 3 | | I | T2N0M0 | Malignant | 2+ | | 3+ | Adjacent normal lung tissue | – | – | | – | NAT | 1+ | 2+ |
| 35 | M | | 69 | Lung | Adenocarcinoma with necrosis | 3 | | I | T2N0M0 | Malignant | 2+ | | 3+ | Adjacent normal lung tissue | – | – | | – | NAT | 1+ | 2+ |
| 36 | F | | 68 | Lung | Adenocarcinoma (sparse) | 3 | | II | T2N1M0 | Malignant | 2+ | | 3+ | Pneumonia | – | – | | – | NAT | 1+ | 2+ |
| 37 | F | | 61 | Lung | Adenocarcinoma | 3 | | I | T2N0M0 | Malignant | 2+ | | 3+ | Emphysema | – | – | | – | NAT | 1+ | 2+ |
| 38 | M | | 62 | Lung | Adenocarcinoma | 3 | | IIIA | T3N0M0 | Malignant | 2+ | | 3+ | Adjacent normal lung tissue | – | – | | – | NAT | 1+ | 1+ |
| 39 | F | | 72 | Lung | Adenocarcinoma | 3 | | II | T2N1M0 | Malignant | 3+ | | 3+ | Adjacent normal lung tissue | – | – | | – | NAT | 1+ | 1+ |
| 40 | M | | 60 | Lung | Adenocarcinoma | 3 | | I | T2N0M0 | Malignant | 2+ | | 3+ | Adjacent normal lung tissue | – | – | | – | NAT | 1+ | 1+ |
| 41 | M | | 49 | Lung | Adenocarcinoma | 3 | | I | T2N0M0 | Malignant | 2+ | | 3+ | Adjacent normal lung tissue | – | – | | – | NAT | 1+ | 1+ |
| 42 | F | | 46 | Lung | Adenocarcinoma | 2 | | I | T1N0M0 | Malignant | 2+ | | 3+ | Adjacent normal lung tissue | – | – | | – | NAT | 1+ | 2+ |
| 43 | M | | 65 | Lung | Adenocarcinoma | 3 | | IIIA | T3N0M0 | Malignant | 2+ | | 3+ | Adjacent normal lung tissue | – | – | | – | NAT | 1+ | 2+ |
| 44 | F | | 62 | Lung | Adenocarcinoma | 3 | | I | T2N0M0 | Malignant | 2+ | | 3+ | Adjacent normal lung tissue | – | – | | – | NAT | 1+ | 2+ |
| 45 | F | | 36 | Lung | Adenocarcinoma | 3 | | IIIA | T2N2M0 | Malignant | 2+ | | 3+ | Adjacent normal lung tissue | – | – | | – | NAT | 1+ | 2+ |
| 46 | M | | 39 | Lung | Adenocarcinoma | 3 | | I | T2N0M0 | Malignant | 3+ | | 3+ | Adjacent normal lung tissue | – | – | | – | NAT | 1+ | 1+ |
| 47 | F | | 58 | Lung | Adenocarcinoma | 3 | | IIIA | T3N0M0 | Malignant | 2+ | | 3+ | Adjacent normal lung tissue | – | – | | – | NAT | 1+ | 2+ |
