# Supplementary material for: Loss of thymidine kinase 1 inhibits lung cancer growth and metastatic attributes by reducing GDF15 expression
Source: PLoS Genet. 2019 Oct 7;15(10):e1008439. doi: 10.1371/journal.pgen.1008439 (PMC6797230; doi:10.1371/journal.pgen.1008439)
Supplement: S2 Table — (DOCX) [file pgen.1008439.s010.docx]

**S2 Table.** Analysis of transcription factors on *TK1* promoter by PROMO 3.0 and rVISTA 2.0.

| **S.No.** | **Transcription factor** | **Predicted binding site on *TK1* promoter**  **(5’-3’ upstream of transcription start)** | **Consensus DNA binding sequence (5’-3’)** |
| --- | --- | --- | --- |
| 1 | AhR | ^-583^ CCACGCCCGG ^-574^ | GCACGCCAGC |
| 2 | Arnt | ^-583^ CCACGCCCGG ^-574^ | GCACGCCAGC |
| 3 | AP-1 | ^-1226^ TGACTGAGC ^-1218^ | GATGAGTCA |
| 4 | AP-2alphaA | ^-459^ GCCTGG ^-454^  ^-1233^ GCCTGG ^-1228^  ^-1410^ GCCTGG ^-1405^ | GCAGGC |
| 5 | AR | ^-1076^ GATCTGTCC ^-1068^ | GCACTGTCC |
| 6 | C/EBPalpha | ^-818^ CATTGTG ^-812^ | GATTGAG |
| 7 | C/EBPbeta | ^-172^ TTGC ^-169^  ^-985^ TTGC ^-982^  ^-1121^ TTGC ^-1118^  ^-1267^ TTGC ^-1264^  ^-1444^ TTGC ^-1441^  ^-1516^ TTGC ^-1513^ | TTGC |
| 8 | c-Myb | ^-66^ AAACTTGG ^-59^ | CAACTGCC |
| 9 | COUP-TF1 | ^-1907^ CCTGAGGTCAAAT ^-1895^ | CGAGAGGTCAAAG |
| 10 | Egr-3 | ^-63^ CTTGGTGGGCGGA ^-51^ | TACACCCACTATA |
| 11 | Elk-1 | ^-1115^ CTTCCTCCC ^-1107^ | CTTCCTCCC |
| 12 | ER-alpha | ^-509^ TATCTG ^-505^  ^-1888^ TATCTG ^-1884^ | TGACC |
| 13 | ETF | ^-237^ GCCCCAGCCCC ^-227^ | GCCCCCCGCAC |
| 14 | GATA-1 | ^-1166^ GAGATA ^-1161^ | TATCTG |
| 15 | GATA-2 | ^-1165^ AGATAAAGC ^-1157^ | GCCCTATCT |
| 16 | GR | ^-387^ CAAAAAT ^-381^  ^-1671^ CAAAAAT ^-1665^ | CAAAAAA |
| 17 | HNF-1 | ^-1173^ GTTAAAAG ^-1166^ | ATATTAAC |
| 18 | HNF-4alpha | ^-237^ TCTCAGCACTTTG ^-227^ | TGTTTGGACTTTG |
| 19 | Ik-1 | ^-1323^ TCCCAGCCACTCG ^-1311^ | TCCCAGCACCTTG |
| 20 | LEF-1 | ^-990^ CTTTGTTG ^-983^ | CTTTGATC |
| 21 | MAZ | ^-223^ GTCCCTCCCTGCA ^-211^ | AACCCTCCCCCCC |
| 22 | NF-1 | ^-533^ TTGGCCAG ^-526^  ^-1665^ TTGGCCAG ^-1658^ | TTGGCCCA |
| 23 | NF-kappaB | ^-167^ TGGGAAACCCAC ^-156^ | GGGAAATTCCCC |
| 24 | NF-Y | ^-1666^ ATTGGCCA ^-1659^ | ATTGGTCA |
| 25 | p53 | ^-312^ CATGCCC ^-306^ | CATGCCC |
| 26 | Pax-5 | ^-32^ CAGGCCC ^-26^  ^-234^ CCAGCCC ^-228^  ^-312^ CATGCCC ^-306^  ^-1524^ GGGCATG ^-1518^  ^-1800^ CAGGCCC ^-1794^ | CAAGCCC |
| 27 | PPAR-alpha | ^-1503^ TAGTCCCAGCT ^-1493^ | CTGACCCAGTG |
| 28 | PR | ^-1870^ TACTGTT ^-1864^ | AACAGTA |
| 29 | PXR-1 | ^-1725^ GGAGTTCA ^-1718^ | TGAACTAA |
| 30 | RBP-Jkappa | ^-171^ TGCTTGGGAAAC ^-160^ | GTTTCCCACGAC |
| 31 | SRY | ^-990^ CTTTGTTGC ^-982^ | AGAACAAAG |
| 32 | STAT4 | ^-266^ ATTTCC ^-261^ | ATTTCC |
| 33 | STAT5A | ^-558^ TTAGTAGAAACGG ^-546^ | TTACCAGAAAAGG |
| 34 | T3R-beta1 | ^-586^ TCACCACGC ^-578^ | TCACCACCG |
| 35 | USF2 | ^-476^ CAGGTGTGAG ^-467^  ^-1026^ GCCACACCTG ^-1017^  ^-1783^ CTCACACCTG ^-1774^ | AGGTCACCTG |
| 36 | VDR | ^-641^ GTTCAAGCG ^-633^ | CGGGTGAAC |
| 37 | XBP-1 | ^-1645^ ATGCCT ^-1640^ | ATGACG |
| 38 | YY1 | ^-7^ CCAT ^-4^  ^-144^ CCAT ^-141^  ^-358^ CCAT ^-355^  ^-538^ CCAT ^-535^  ^-994^ CCAT ^-991^  ^-1558^ CCAT ^-1553^  ^-1581^ CCAT ^-1578^  ^-1689^ CCAT ^-1686^  ^-1893^ CCAT ^-1890^  ^-1949^ CCAT ^-1946^ | CCAT |
