# Supplementary material for: Loss of thymidine kinase 1 inhibits lung cancer growth and metastatic attributes by reducing GDF15 expression
Source: PLoS Genet. 2019 Oct 7;15(10):e1008439. doi: 10.1371/journal.pgen.1008439 (PMC6797230; doi:10.1371/journal.pgen.1008439)
Supplement: S3 Table — (DOCX) [file pgen.1008439.s011.docx]

**S3 Table.** Fold change for significantly altered genes in A549 cells expressing *TK1* shRNAs compared to the cells expressing non-silencing shRNA.

| **Gene Symbol** | ***TK1* shRNA#1** | | | ***TK1* shRNA#2** | | |
| --- | --- | --- | --- | --- | --- | --- |
|  | **Fold change (FC)** | **Log fold change (logFC)** | **Adj. p-value** | **Fold change (FC)** | **Log fold change (logFC)** | **Adj. p-value** |
| ABCA1 | 1.65 | 0.72 | 1.02E-06 | 0.60 | -0.73 | 1.14E-06 |
| ANG | 0.59 | -0.75 | 2.84E-08 | 1.51 | 0.59 | 3.83E-06 |
| ANG | 0.59 | -0.75 | 6.90E-08 | 1.58 | 0.66 | 3.92E-08 |
| C2orf82 | 1.60 | 0.68 | 1.99E-07 | 1.72 | 0.78 | 3.12E-08 |
| CAV2 | 1.67 | 0.74 | 2.57E-08 | 1.64 | 0.71 | 4.68E-08 |
| CD24 | 1.73 | 0.79 | 4.40E-09 | 2.01 | 1.00 | 7.72E-11 |
| FGB | 0.65 | -0.61 | 4.17E-06 | 2.55 | 1.35 | 7.72E-11 |
| FGG | 0.58 | -0.78 | 1.04E-05 | 1.72 | 0.78 | 1.34E-05 |
| GDF15 | 0.66 | -0.59 | 4.31E-07 | 0.66 | -0.59 | 5.32E-07 |
| HILPDA | 0.66 | -0.59 | 6.47E-06 | 0.64 | -0.62 | 4.54E-06 |
| HIPK2 | 0.60 | -0.71 | 9.92E-09 | 0.56 | -0.82 | 1.33E-09 |
| HMGB3 | 0.51 | -0.96 | 4.95E-09 | 0.62 | -0.68 | 5.67E-07 |
| KRT19 | 1.61 | 0.69 | 1.00E-06 | 2.07 | 1.05 | 3.32E-09 |
| KRT4 | 0.63 | 0.64 | 2.40E-06 | 2.23 | 1.16 | 8.81E-10 |
| MMD | 0.56 | -0.82 | 4.95E-09 | 0.54 | -0.86 | 2.10E-09 |
| NTS | 0.65 | -0.61 | 4.38E-07 | 1.87 | 0.90 | 2.10E-09 |
| SERPINE1 | 1.96 | 0.97 | 4.95E-09 | 1.68 | 0.75 | 1.86E-07 |
| TAGLN | 1.80 | 0.85 | 4.00E-09 | 1.65 | 0.73 | 2.21E-08 |
| TK1 | 0.37 | -1.40 | 9.40E-13 | 0.48 | -1.05 | 5.37E-11 |
