# Supplementary material for: Loss of thymidine kinase 1 inhibits lung cancer growth and metastatic attributes by reducing GDF15 expression
Source: PLoS Genet. 2019 Oct 7;15(10):e1008439. doi: 10.1371/journal.pgen.1008439 (PMC6797230; doi:10.1371/journal.pgen.1008439)
Supplement: S4 Table — (DOCX) [file pgen.1008439.s012.docx]

**S4 Table.** Primer sequences for RT-qPCR analysis; clone ID and catalog numbers for shRNAs (Open Biosystems); antibodies used; source and concentration of chemical inhibitors used.

| **Application** | **Gene symbol** | **Forward primer (5′-3′)** | **Reverse primer (5′-3′)** |
| --- | --- | --- | --- |
| **RT-qPCR** | TK1 | ggggcagatccaggtgattc | ccatggtgttccggtcatgt |
|  | AhR | gcagcgccaacatcacctac | ggctagccaaacggtccaac |
|  | Arnt | aggataaggggtgggggaga | agcaggggaacagccagaag |
|  | AP-1 | ttcaggaggctggaggaagg | tggtcacagcacatgccact |
|  | AP-2 alphaA | ttacctcacgccatcgagga | ggcattgctgttggacttgg |
|  | AR | ggcggggtaagggaagtagg | cccgggttctggatcacttc |
|  | C/EBP alpha | tcccagagggaccggagtta | cattgcacaaggcactgctg |
|  | C/EBP beta | ggacaagcacagcgacgagt | cttgtgctgcgtctccaggt |
|  | c-Myb | acggtccgaaacgttggtct | tgcgatttctgcccatctgt |
|  | COUP-TF1 | aactgtcccatcgaccagca | gtgcgtactggcctggattg |
|  | Egr-3 | gtggctggaggaggtggtct | tcagcgatggctgctctttc |
|  | Elk-1 | ggccatactcaggggtccag | cctccctggccaaaaagttg |
|  | ER-alpha | agaacagcctggccttgtcc | tgaaccagctccctgtctgc |
|  | ETF | actgatcgcccgctacatca | ctgggcagaggacatggttg |
|  | GATA-1 | ggggttttcttcccctctgg | ggcctcagcgtccctgtagt |
|  | GATA-2 | ttcagccactccggacacat | tgggctgctaagggtttggt |
|  | GR | acaggcagcgatggtctcag | ctgggtcagagcctcagcaa |
|  | HNF-1 | aagaagccttccggcacaag | gccgctgcttgagggtactt |
|  | HNF-4alpha | atcgtcaagcccctctctgc | tggtcctggcatcacgtctt |
|  | Ik-1 | atatggttccccaccgacca | ctcgcgtttgttttggcaag |
|  | LEF-1 | ccaggctggtctgcaagaga | tgcacgttgggaatgagctt |
|  | MAZ | caaggggagcaggaggaaga | gaagaggggagggggacact |
|  | NF-1 | ccatggtcctctcccaaagg | gagaaggttgccccatgtcc |
|  | NF-kappaB | accacctctcaggcccactc | tttgcggaaggatgtctcca |
|  | NF-Y | ccttacggtcgctgggaatc | gccagggcaggtctactgct |
|  | p53 | accggagtcattgggaagga | gccttctctgtcccccagaa |
|  | Pax-5 | aatggcgcccttcttttcaa | ttatggaggggaaggccaga |
|  | PPARA | ccaggagaacatgcccacag | acacccagcaggccctacat |
|  | PR | ccaaggcagagctcaggtca | gccagcagtcctgcaacagt |
|  | PXR-1 | tggcctgttgggttgaattg | accgctgcagtgatgacaca |
|  | RBP-J kappa | tgcattccgagaaggttgga | gattcgttagggggcacctg |
|  | SRY | atcccgcttcggtactctgc | ggcctagctggtgctccatt |
|  | STAT4 | ttcccacaaaagcagctcca | ttccctgccaccttgctgta |
|  | STAT5A | caagagtgcgccgagtctgt | acctgtctcccctccccttc |
|  | T3R-beta1 | gtcgtcgccacatctcatcc | tggaaggtctgggcacttga |
|  | USF2 | cagcaagacgggagcgagta | tgcctcaggagctcgttgtc |
|  | VDR | cccaagctgtctgaggagca | ggctccctccaccatcattc |
|  | XBP-1 | cccatggattctggcggtat | ctggggaagggcatttgaag |
|  | YY1 | cccagggcaggaatgaaaag | aagcgtttcccacagccttc |
|  | GDF15 | tccggatactcacgccagaa | tctggcaaggctgagctgac |
|  | DCK | tgttgccagatggtgcaatg | tgccattcagagaggcaagc |
|  | DGUOK | cacccaaaaagcctgcactg | ttctcagggaagggctccag |
|  | DTYMK | acacgccagggtctcctctc | agggagggagagagggtgct |
|  | NME1 | gcccgaaggaggaagagaca | tccctccttgctctgtgctg |
|  | HMGB3 | tgttgccagcctcgtcaaat | ctgctcccccacttctgctt |
|  | MMD | gaacttggacccctggcatc | atgtcaccaccaaggctgga |
|  | HILPDA | caacacagagcccaccaagg | cggtgctcagcttgtctgct |
|  | HIPK2 | cggggaatgtcgagtcactg | ctcactgtgcccagcagctt |
|  |  |  |  |
|  |  |  |  |
|  | ACTINB | gcatggagtcctgtggcatc | ttctgcatcctgtcggcaat |
|  | | | |
| **ChIP** |  | **Forward primer** | **Reverse primer** |
|  | TK1  promoter MAZ  binding | CTCTGCATGCCCACAGGAGT | TGGGTTTCCCAAGCAAGGTT |
|  | ACTINB | GAGGGGAGAGGGGGTAAAAA | AAAGGCGAGGCTCTGTGCT |
|  | | | |
| **shRNAs** | **Gene symbol** | **Clone ID** | **Catalog number** |
|  | *TK1* | RHS3979-9631265 | TRCN0000010135 |
|  | *TK1* | RHS3979-9631268 | TRCN0000010127 |
|  | *MAZ* | RHS3979-9583032 | TRCN0000015343 |
|  | *MAZ* | RHS3979-9583035 | TRCN0000015346 |
|  | *DCK* | RHS3979-9631046 | TRCN0000009932 |
|  | *DCK* | RHS3979-9631048 | TRCN0000009934 |
|  | *DGUOK* | RHS3979-9574937 | TRCN0000006074 |
|  | *DGUOK* | RHS3979-9574940 | TRCN0000006076 |
|  | *DTYMK* | RHS4430-101103458 | V3LHS_366125 |
|  | *DTYMK* | RHS4430-101106747 | V3LHS_366127 |
|  | *NME1* | RHS3979-9631176 | TRCN0000010063 |
|  | *NME1* | RHS3979-9631177 | TRCN0000010064 |
|  | *GDF15* | RHS3979-9625575 | TRCN0000058391 |
|  | *GDF15* | RHS3979-9625576 | TRCN0000058392 |
|  | *HMGB3* | RHS3979-9586282 | TRCN0000018518 |
|  | *HMGB3* | RHS3979-9586284 | TRCN0000018520 |
|  | *MMD* | RHS3979-9630172 | TRCN0000062988 |
|  | *MMD* | RHS3979-9630176 | TRCN0000062992 |
|  | *HILPDA* | RHS4430-101098607 | V3LHS_367042 |
|  | *HILPDA* | RHS4430-101102577 | V3LHS_367045 |
|  | *HIPK2* | RHS3979-9571833 | TRCN0000003202 |
|  | *HIPK2* | RHS3979-9571834 | TRCN0000003203 |
|  |  |  |  |
| **Plasmids** | **Name** | **Source** | **Catalog number** |
|  | LentiORF-GDF15 | GE Dharmacon | OHS6085-213574818 |
|  | GST-RBD | Addgene | 15247 |
|  | | | |
| **Immunoblotting** | **Protein symbol** | **Antibody source** | **Dilution** |
|  | TK1 | Cell signaling (Cat# #8960) | 1:1000 |
|  | Phospho-ERK | Cell signaling (Cat# #4376) | 1:1000 |
|  | Total-ERK | Cell signaling (Cat# #4695) | 1:1000 |
|  | MAZ | Proteintech (Cat# 21068-1-AP) | 1:1000 |
|  | RhoA | Cell signaling (Cat# #2117) | 1:1000 |
|  | GST | Santa Cruz Biotechnology (Cat# sc-138) | 1:1000 |
|  | DCK | ABclonal (Cat# A0185) | 1:1000 |
|  | DGUOK | ABclonal (Cat# A6898) | 1:1000 |
|  | DTYMK | ABclonal (Cat# A6370) | 1:1000 |
|  | NME1 | ABclonal (Cat# A0259) | 1:1000 |
|  | GDF15 | ABclonal (Cat# A0185) | 1:1000 |
|  | V5 | Cell signaling (Cat# #13202) | 1:1000 |
|  | ACTINB | Cell signaling (Cat# #8457) | 1:2500 |
|  |  |  |  |
| **Immunohistochemistry** | **Protein symbol** | **Antibody source** | **Dilution** |
|  | TK1 | Invitrogen (Cat# PA5-29686) | 1:500 |
|  |  |  |  |
| **Immunofluorescence staining** | **Protein symbol** | **Antibody source** | **Dilution** |
|  | Vinculin | EMD Millipore (Cat# MAB3574) | 1:200 |
|  | Phospho-γ-H2AX | Cell signaling (Cat# 9718) | 1:200 |
|  | | | |
| **Pharmacological treatment** | **Compounds** | **Concentration** | **Source** |
|  | Trametinib | Indicated concentrations | Calbiochem |
